# Comparative effectiveness of adjuvant treatment for hepatocellular carcinoma with high risk of recurrence: A systematic review and network meta-analysis

**DOI:** 10.1371/journal.pone.0335457

**Published:** 2025-12-04

**Authors:** Zha Peng, Leigu Shen, Yaqiong Wang, Boyu Chen, Zhuangrong Zhu, Chengyi He, Hai Huang

**Affiliations:** 1 Department of Surgery, Dongguan Hospital of Guangzhou University of Traditional Chinese Medicine, Dongguan, China; 2 Department of General Surgery, The Second Affiliated Hospital of Guangxi Medical University, Nanning, China; 3 Department of Hepatobiliary Surgery, Wuming Hospital of Guangxi Medical University, Nanning, China; 4 Department of General Surgery, The First People’s Hospital of Loudi, Loudi, China; Sichuan University, CHINA

## Abstract

**Objective:**

To identify the most effective postoperative intervention regimen for persons with hepatocellular carcinoma (HCC) at high risk of recurrence.

**Methods:**

A network meta-analysis was conducted by collecting eligible studies from Medline, Embase, the Cochrane Central Register of Controlled Trials, and Web of Science databases. Pairwise and network meta-analyses were applied to pooled data on overall survival (OS) and recurrence-free survival (RFS).

**Results:**

35 studies involving 6,372 patients were analyzed. Pairwise meta-analysis showed higher OS (HR = 0.64, 95% CI: 0.56–0.72; *P* < 0.001; *I*^2^ = 70.9%, random effects model) and RFS (HR = 0.62, 95%CI 0.56–0.70; *P* < 0.001; *I*^2^ = 60.2%, random effects model) in the adjuvant intervention group compared to surgery alone. ICIs&TKIs (SUCRA = 87.3%) was significantly more effective for OS, and TKIs&TACE (SUCRA = 84.1%) was significantly more effective for RFS.

**Conclusions:**

Reasonable postoperative intervention regimens can reduce recurrence risk and improve prognosis. ICIs&TKIs and TKIs&TACE are likely the two most effective adjuvant intervention regimens for individuals with HCC who are at high risk of recurrence.

## Introduction

Hepatocellular carcinoma (HCC) is the sixth most common malignant tumor and the fourth leading cause of cancer-related deaths worldwide, posing a serious threat to human health [1]. Surgery is the primary treatment for primary liver cancers [2]. However, the cumulative recurrence rate after hepatectomy is as high as 60–70% [3], and the 5-year survival rate is less than 50% [4]. Therefore, taking effective measures to reduce the postoperative recurrence of HCC is key to improving prognosis and prolonging survival.

It is generally believed that narrow resection margins (<1.0 cm) or positive resection margins, multiple tumors, presence of satellite lesions, tumor diameter >5 cm, vascular invasion, bile duct invasion, and poor tumor differentiation are high-risk factors for early recurrence after radical resection of HCC [5–8]. HCCs with these characteristics are often more aggressive, suggesting that patients have a higher recurrence rate and shorter survival time. An earlier study has found that two-thirds of patients with microvascular invasion (MVI) recur within two years [9]. With the development of multidisciplinary treatment methods for HCC, an increasing number of studies have explored effective adjuvant intervention strategies. These strategies aimed to reduce recurrence rates and prolong survival in HCC patients with high-risk recurrence factors. The adjuvant intervention strategies included transcatheter arterial chemoembolization (TACE), hepatic artery infusion chemotherapy (HAIC), radiotherapy (RT), targeted therapy, immunotherapy, and combination regimens [2,10–13]. Many studies have confirmed that adjuvant intervention for such patients can reduce postoperative recurrence rate and prolong survival [2,10–13]. However, there were relatively few head-to-head studies directly comparing the efficacy of different adjuvant intervention regimens, and the case inclusion criteria of various studies were highly heterogeneous. As a result, there is no consensus on the selection of adjuvant intervention strategies for patients with high-risk recurrence risk factors after surgery, and more reliance is placed on clinicians’ empirical choices. Previous network meta-analysis [14] has compared the efficacy of adjuvant HAIC, RT, TACE, and sorafenib in HCC patients with high-risk recurrence risk factors. The results showed that adjuvant sorafenib and RT can effectively improve overall survival (OS) and disease-free survival (DFS) in patients undergoing radical resection of HCC with a high risk of recurrence. However, Xu et al.’s [14] study included only a single intervention measure and did not include a combined intervention regimen. Indeed, some clinicians are exploring various combination therapeutic regimens, such as immune checkpoint inhibitors (ICIs) plus tyrosine kinase inhibitors (TKIs) (ICIs&TKIs), and TKIs combined with TACE (TKIs&TACE), and their real-world clinical efficacy needs to be thoroughly evaluated.

This network meta-analysis employed a Bayesian framework to systematically compare the therapeutic efficacy of postoperative interventions in HCC patients exhibiting elevated recurrence risk profiles (e.g., microvascular invasion, multifocal lesions), with the ultimate goal of establishing an evidence-based hierarchy for adjuvant therapy selection in clinical practice.

### Study design and methods

To evaluate optimal treatment strategies for HCC patients with high-risk factors to improve prognosis, we designed a network meta-analysis. The analyzed postoperative therapeutic strategies included surgery alone, monotherapy with TKIs, Aspirin, ICIs, RT, TACE or HAIC, as well as combination therapies such as ICIs&TKIs and TKIs&TACE. This network meta-analysis was reported following the PRISMA 2020 [15], and has been registered in the PROSPERO database (registration number: CRD42024586032).

### Search strategy and inclusion criteria

1) Literature search: we systematically searched Medline, Embase, Web of Science, and the Cochrane Central Register of Controlled Trials for all Randomized control Trails (RCTs), prospective cohort studies and retrospective studies using propensity score matching (PSM) analysis related to our study from inception to 23/09/2024. The publication language of all studies was English. Search strategies consisted of subject words and free words. The full search strategy is presented in Suplementary material S1 File. In addition, we manually searched relevant reviews and references in the included literature to expand the research sample size as much as possible;
2) Participants: patients diagnosed with HCC who underwent radical liver resection and had at least one high-factor for recurrence. In this trial, high recurrence risk was defined as multiple tumors (≥2), satellite lesions, tumor diameter >5 cm, MVI, portal vein tumor thrombosis (PVTT), narrow or positive margins.
3) Intervention: the postoperative adjuvant therapies identified in the literature comprised: postoperative observation only (surgery alone), Aspirin, TACE, TKIs monotherapy, ICIs, HAIC, RT and Combination therapies such as ICIs&TKIs or TKIs&TACE. Eligible studies were searched to compare the prognosis of any two interventions through head-to-head or indirect comparisons.
4) Outcome factors: the included studies must report at least one of the following outcomes: OS, DFS, or RFS (recurrence-free survival), along with the hazard ratio (HR) and its 95% confidence interval (CI). OS was defined as the time from radical surgery to death or the last follow-up. DFS was defined as the time from radical surgery to recurrence. RFS was defined as the time from a specific treatment or event to the disease recurrence or the occurrence of a specific outcome event. In this study, RFS and DFS were regarded as the same outcome variable. HR refers to the ratio of hazard rates between the intervention and control groups and represents the time of event occurrence in tumor studies. An intervention with a high HR was associated with poor outcomes (death or recurrence).

### Literature quality assessment and data extraction

The two authors (Z.P. and L.G.S.) independently conducted risk of bias assessments for the included RCTs in this study using the Cochrane risk-of-bias tool, version 2 (RoB 2). For non-randomized controlled studies, the Risk of Bias in Non-Randomised Studies – of Interventions (ROBINS-I) tool was employed to evaluate the risk of bias. The certainty of evidence in RCTs was assessed using the Grading of Recommendations, Assessment, Development, and Evaluations (GRADE) approach [16].

Two independent researchers (Y.Q.W and B.Y.C.) conducted data extraction after reading the full text. Discrepancies were resolved by discussion or, if necessary, by third researcher (C.Y.H.) adjudication. The extracted data included: 1) first author, publication time, study type, begin and end of study, crowd characteristics, treatment, and sample size; 2) patient-related information such as gender, age, number of tumors, tumor size, liver cirrhosis, MVI, hepatitis B virus or hepatitis C virus infection, Edmondson’s grading, resection margin status; 3) outcome information includes HR and 95%CI of OS, DFS, or RFS after different intervention measures. HR and 95%CI were directly extracted from the article or extracted from the Kaplan-Meier curve using Engauge Digitizer software (version 4.1, M Mitchell) [17]. For studies reporting median follow-up duration and *P*-values, HRs may be calculated via validated web-based statistical platforms (https://ebm-helper.cn/Conv/HR.html).

### Statistical methods

We combined all direct and indirect evidence to compare the efficacy among different treatment strategies and reported them as HR for OS and RFS and the corresponding 95%CI. A combined HR < 1 indicated better efficacy in the treatment group. If the 95%CI of the combined HR did not overlap with 1, the difference was considered to be statistically significant. First, traditional pairwise meta-analyses were performed using Stata software (version 15.0), directly comparing the treatment group with the surgery alone group. A random effects model was used, and the *I*^2^ statistic and *P*-value were used to evaluate the heterogeneity. Second, the “gemtc” (version 0.8–8) and “JAGS” (version 4.3.0) in R (version 4.0.30) were used to construct the network node diagram. Third, network meta-analyses were performed using the Bayesian framework in R. Consistency and inconsistency modeling were performed on the network nodes. The difference in deviance information criteria (DIC) <5 indicates that the data basically meet the premise of consistency [17]. The convergence of iterations was evaluated using Brooks-Gelman-Rubin statistics and trace plots. In the Bayesian method, the probability of each intervention becoming the most effective treatment method was calculated using the area under the cumulative ranking curve (SUCRA). For each outcome, the larger the SUCRA value, the better the ranking of a certain therapy among various treatment methods. Bayesian *P*-value generated by the node-splitting method was used to verify the consistency of direct and indirect comparisons. Publication bias was evaluated by observing the symmetry of the funnel plots.

## Results

### Characteristics of included studies and bias assessment

A total of 3251 articles were initially retrieved. After removing duplicate articles and screening by title and abstract, 164 potentially eligible articles were reviewed in full text. Finally, 33 eligible articles were identified. In addition, two articles were supplemented by manual search. A total of 35 articles involving 6,372 patients were included in the final analysis (S2 File), comprising 11 RCTs [2,11,18–26], 7 prospective cohort studies [10,12,27–31], and 17 retrospective studies [13,32–47]. The baseline characteristics of the included studies are reported in S3 File. Thirty one of the studies used a composite endpoint combining OS with DFS or RFS. Three studies used only OS [19,25,46], and one study used only DFS as endpoint [34] (Table 1).

**Table 1:** Outcome information of included studies.

Study	Year	PMID	treatment	Control	OS(HR,95%CI)	RFS/DFS((HR,95%CI)
Xiang [27]	2024	37812183	TACE	Hepatectomy alone	0.527(0.2896,0.959)	0.408(0.2216,0.7513)
Peng [26]	2024	38568599	Sorafenib+TACE	Sorafenib	0.57 (0.36–0.91)	0.57 (0.39–0.83)
Bai [2]	2024	38488934	RT	TACE	0.73 (0.22–2.37)	0.78 (0.3–2.0)
Wang [32]	2023	38242982	sintilimab	Hepatectomy alone	0.505 (0.254–1.006)	0.534 (0.360–0.792)
Lu [19]	2023	37810112	Aspirin	Hepatectomy alone	0.664(0.419–1.052)	NR
Luo [47]	2023	36905230	TACE	Hepatectomy alone	0.43 (0.18–0.99)	0.54 (0.31–0.92)
Long [10]	2023	36634853	RT	Hepatectomy alone	0.80 (0.26–2.43)	0.57 (0.28–1.15)
Li [12]	2023	37452107	ICIs alone or with TKIs	Hepatectomy alone	0.31 (0.17–0.59)	0.52 (0.35–0.76)
Li [32]	2023	37359534	ICIs + TKIs	Hepatectomy alone	0.32 (0.12–0.86)	0.47 (0.23–0.96)
Bai [33]	2023	37029989	TACE	Hepatectomy alone	0.63 (0.33–1.21)	0.66 (0.38–1.16)
Li [11]	2023	36525610	HAIC	Hepatectomy alone	0.64 (0.36–1.14)	0.59 (0.43–0.81)
Gou [35]	2022	35643251	RT	Hepatectomy alone	0.72 (0.5429–0.9657)	0.6434 (0.458–0.9039)
Lin [34]	2022	35300207	TKIs+TACE	TACE	NR	0.67(0.38–1.16)
Qiu [13]	2022	35795039	TACE	Hepatectomy alone	0.5541 (0.3261–0.9413)	0.4579 (0.2724–0.7697
Wang [37]	2021	33455865	TACE	Hepatectomy alone	0.4762 (0.3061–0.7408)	0.1474 (0.0471–0.4611)
Li [38]	2021	34631511	sorafenib	Hepatectomy alone	0.7647 (0.5984–0.9772)	0.7857 (0.3463–1.7828)
Wang [36]	2021	32440804	TACE	Hepatectomy alone	0.7506 (0.5178–1.0882)	0.7468 (0.5947–0.9378)
Huang [40]	2020	33061610	TACE	Hepatectomy alone	0.5294 (0.334–0.8391)	0.25 (0.0958–0.6521)
Rong [20]	2020	33223759	RT	Hepatectomy alone	0.85 (0.48–1.50)	0.62 (0.33–1.14)
Wang [28]	2020	32611327	RT	Hepatectomy alone	0.6675 (0.5069–0.879	0.2456 (0.0902–0.6686)
Wang [39]	2020	32547217	TACE	Hepatectomy alone	0.625 (0.4313–0.9056)	0.884 (0.8096–0.9653)
Zhang [41]	2019	30767178	TACE	Hepatectomy alone	0.6272 (0.4565–0.8617)	0.7702 (0.6268–0.9464)
Zhang [42]	2019	31153833	sorafenib	Hepatectomy alone	0.806 (0.6891–0.9428)	0.4688 (0.2985–0.7361)
Wang [44]	2019	30863091	RT	TACE	0.62 (0.26–1.49)	0.47 (0.27–0.83)
Sun [21]	2019	31176205	RT	Hepatectomy alone	0.444(0.243–0.813)	0.358(0.197–0.652)
Qi [29]	2019	30103903	TACE	Hepatectomy alone	0.60 (0.13–2.76)	0.99 (0.53–1.86)
Wang [43]	2019	30249510	TACE	Hepatectomy alone	0.519 (0.323–0.832)	0.655 (0.450–0.954)
Wei [22]	2018	30305149	TACE	Hepatectomy alone	0.68 (0.48–0.97)	0.70 (0.52–0.95)
Wang [23]	2018	29420221	TACE	Hepatectomy alone	0.59 (0.36–0.97)	0.68 (0.49–0.93)
Li [45]	2017	28032575	TACE	Hepatectomy alone	0.66 (0.35–1.23)	0.78 (0.46–1.34)
Hsiao [30]	2017	28728985	HAIC	Hepatectomy alone	1.0089 (0.9533–1.0676)	1.0771 (0.3182–3.6459)
Xia [31]	2016	27340354	sorafenib	Hepatectomy alone	0.72 (0.5511–0.9406)	0.8333 (0.704–0.9863)
Li [46]	2015	24972992	TACE	Hepatectomy alone	0.4286 (0.1967–0.9339)	NR
Zhong [24]	2009	19408012	TACE	Hepatectomy alone	0.72 (0.47–1.10)	0.55 (0.37–0.82)
Peng [25]	2009	19285298	TACE	Hepatectomy alone	0.61(0.30–0.98)	NR

OS, overall survival; DFS, disease-free survival; RFS, recurrence-free survival; HR, hazard ratio; CI, confidence intervals; TACE, transhepatic arterial chemoembolization; HAIC, hepatic artery infusion chemotherapy; RT, radiotherapy; ICIs, immune checkpoint inhibitors; TKIs, Tyrosine kinase inhibitors; ICIs&TKIs, ICIs combined with TKIs; TKIs&TACE, TKIs combined with TACE; MVI, microvascular invasion; PVTT, portal vein tumor thrombosis; NR, not reported.

The GRADE assessment for OS and RFS is shown in the S4 File. The initial certainty of evidence for all RCTs was rated as moderate. Of the 35 studies included in the network meta-analysis, the majority were assessed as having moderate risk of bias, and no study had high risk of bias (S5 and S6 File).

### Pairwise meta-analysis

**Fig 1:**
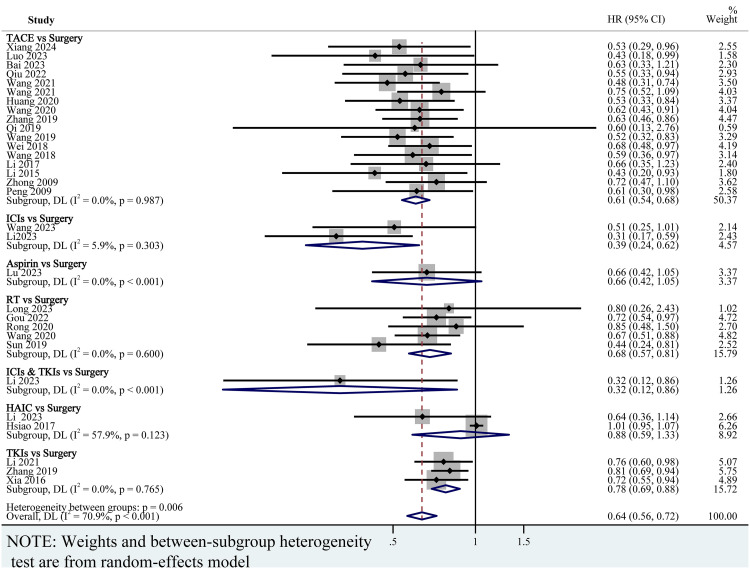
Forest plot of OS for pairwise meta-analyses. TACE, transhepatic arterial chemoembolization; HAIC, hepatic artery infusion chemotherapy; RT, radiotherapy; ICIs, immune checkpoint inhibitors; TKIs, Tyrosine kinase inhibitors; ICIs&TKIs, ICIs combined with TKIs.

**Fig 2:**
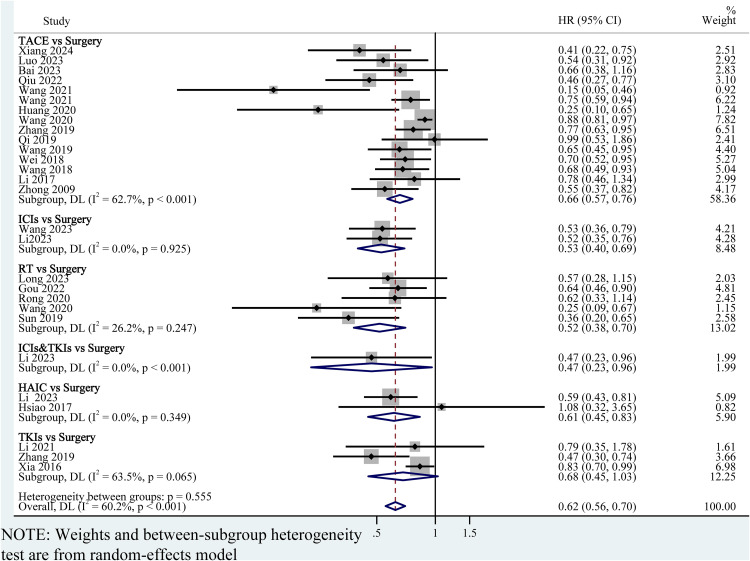
Forest plot of RFS for pairwise meta-analyses. TACE, transhepatic arterial chemoembolization; HAIC, hepatic artery infusion chemotherapy; RT, radiotherapy; ICIs, immune checkpoint inhibitors; TKIs, Tyrosine kinase inhibitors; ICIs&TKIs, ICIs combined with TKIs.

To determine whether postoperative adjuvant therapies improve clinical outcomes compared with surgery alone, we performed pairwise meta-analyses. Among all the included studies, 31 studies reported the original HR and 95%CI for OS (Fig 1), and 29 studies reported the original HR and 95%CI for RFS (Fig 2). The overall HR and 95% CI for OS and RFS were 0.64 (95% CI: 0.56–0.72; P = 0.006; *I*^2^ = 70.9%, random effects model) (Fig 1) and 0.62 (95%CI: 0.56–0.70; P = 0.555; *I*^2^ = 60.2%, random effects model) (Fig 2) respectively, indicating that the adjuvant group has better efficacy than the surgery alone group. Compared with the surgery alone group, TACE [0.61, (0.54–0.68)], ICIs [0.39 (0.24, 0.62)], RT [0.68 (0.57, 0.81)], ICIs&TKIs [0.32 (0.12, 0.86)] and TKIs [0.78 (0.69, 0.88)] was associated with prolonged survival (Fig 1). The point estimate for OS suggested a potential benefit for postoperative aspirin [0.66, (0.42,1.05)] or HAIC [0.88, (0.59,1.33)] compared to surgery alone; however, the confidence intervals were wide and the result was not statistically significant (Fig 1).

For RFS, the pooled HRs strongly support that postoperative adjuvant administration of TACE [0.66 (0.57, 0.76)], ICIs [0.53 (0.40, 0.69)], RT [0.52 (0.38, 0.70)], HAIC [0.61 (0.45, 0.83)] and ICIs&TKIs [0.47 (0.23, 0.96)] can significantly reduce the recurrence risk of patients (Fig 2). Although adjuvant therapy with TKIs postoperatively was associated with a numerical improvement in RFS compared to the surgery-alone group [0.68 (0.45, 1.03)], the effect did not reach statistical significance (Fig 2).

### Network meta-analysis

**Fig 3:**
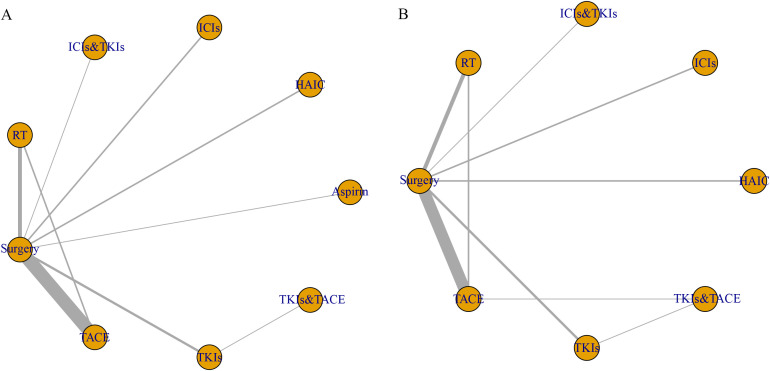
Network diagram of eligible comparisons for OS (A) and RFS (B). Each circular node represents a variety of interventions. The width of lines between the nodes is proportional to the number of trials performing head-to-head comparisons. TACE, transhepatic arterial chemoembolization; HAIC, hepatic artery infusion chemotherapy; RT, radiotherapy; ICIs, immune checkpoint inhibitors; TKIs, Tyrosine kinase inhibitors; ICIs&TKIs, ICIs combined with TKIs; TKIs&TACE, TKIs combined with TACE.

We constructed network diagrams for OS and RFS based on the included literatures (Fig 3). These diagrams clearly demonstrate the evidence structure: surgery alone is the dominant comparator, with substantial direct evidence available for comparing it against interventions like postoperative TACE, RT, ICIs, HAIC, and TKIs. This abundance of direct head-to-head comparisons enables robust pairwise effect assessment for most of these interventions. However, direct evidence for pairwise comparisons not involving surgery alone (such as RT vs. TACE) is sparse. Additionally, as showed in Panel A (Fig 3), no direct comparison trials were identified between the combination therapy TKIs&TACE and HAIC alone. Therefore, while our analysis leverages the extensive direct evidence elsewhere, the estimation of the relative effect between TKIs&TACE and HAIC alone in OS will rely exclusively on indirect evidence derived from the network.

**Fig 4:**
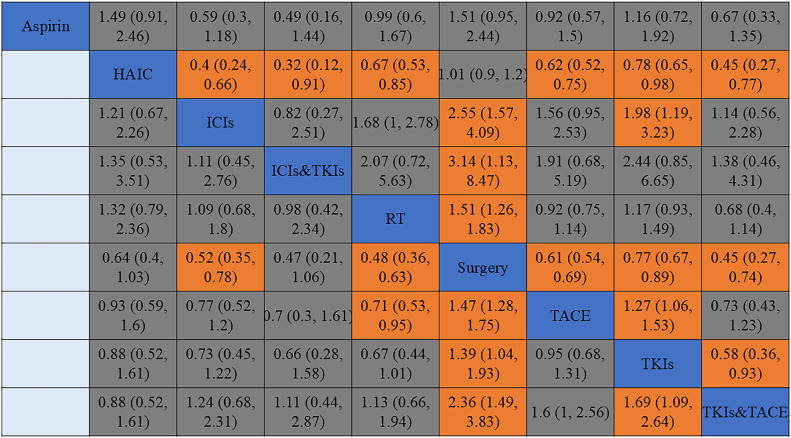
Pooled estimates of the network meta-analysis for OS and RFS. Values in each cell show HR (95%CI) for the comparison between the column defining intervention and the row defining intervention for OS (above the diagonal) and RFS (below the diagonal). HR less than 1 favor the treatment in the corresponding column. Orange indicates significant difference. TACE = transhepatic arterial chemoembolization, HAIC = hepatic artery infusion chemotherapy, RT = radiotherapy, ICIs = immune checkpoint inhibitors, TKIs = Tyrosine kinase inhibitors, ICIs&TKIs = ICIs combined with TKIs, TKIs&TACE = TKIs combined with TACE.

The network meta-analysis evaluating OS and RFS in HCC patients at high recurrence risk was conducted. The effects of postoperative interventions were quantified using HRs with 95%CIs. For OS (Fig 4), results showed that efficacy of postoperative ICIs, ICIs&TKIs, RT, TACE, TKIs or TKIs&TACE was better than that of HAIC. The efficacy of adjuvant ICIs was better than that of TKIs. The efficacy of TACE or TKIs&TACE was better than that of TKIs, and there was no significant difference in the efficacy between postoperative TACE and ICIs.

For RFS (Fig 4), although the efficacy of TKIs&TACE was better than that of TKIs alone, there was no significant difference in efficacy compared with TACE alone. The efficacy of postoperative RT was better than that of TACE, but there was no significant difference in efficacy compared with other adjuvant treatment regimens. In addition, there was no significant difference in efficacy among HAIC, ICIs, ICIs&TKIs, and TKIs.

**Fig 5:**
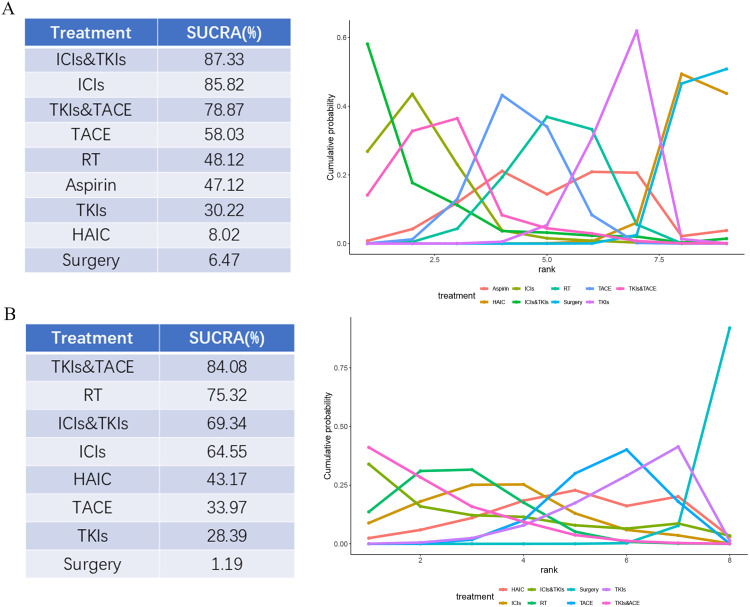
Surface under the cumulative ranking curve (SUCRA) values for OS (A) and RFS (B). TACE = transhepatic arterial chemoembolization; HAIC = hepatic artery infusion chemotherapy; RT = radiotherapy; ICIs = immune checkpoint inhibitors; TKIs = Tyrosine kinase inhibitors; ICIs&TKIs = ICIs combined with TKIs; TKIs&TACE = TKIs combined with TACE.

The surface under the cumulative ranking curve (SUCRA) values for OS and RFS was presented in Fig 5. The SUCRA analysis results indicated that for OS (Fig 5A), the most effective intervention was ICIs&TKIs, followed by ICIs, TKIs&TACE, TACE, RT, Aspirin, TKIs, HAIC and surgery alone. For RFS (Fig 5B), the most effective intervention was TKIs&TACE, followed by RT, ICIs&TKIs, ICIs, HAIC, TACE, TKIs and surgery alone.

The consistency of the model was evaluated using density trace plots (S7 and S8 Files) and Brooks-Gelman-Rubin diagnostic plots (S9 and S10 Files). Single chain fluctuations were not visible to the naked eye, and the density map was normally distributed, suggesting an excellent convergence for these models. For OS and RFS, heterogeneity tests showed that the *I*^2^ of most studies was lower than 50%, suggesting small heterogeneity (S11 and S12 Files). The Bayesian *P*-value showed no obvious inconsistency (S13 and S14 Files). The funnel plot, which was used to assess publication bias, showed general symmetry among the included studies, indicating a minimal risk of publication bias (S15 File).

## Discussion

HCC is a highly malignant tumor characterized by a significant postoperative recurrence rate, primarily due to the presence of numerous high-risk recurrence factors among patients. Despite recent advancements in surgical techniques, perioperative care, and patient selection, which have collectively reduced surgical complications and mortality, the persistent high recurrence rate poses a considerable challenge [1]. This underscores the need for effective adjuvant treatment strategies. This network meta-analysis systematically evaluates the clinical efficacy of various adjuvant interventions following radical resection of HCC, providing valuable insights for clinical decision-making and future research endeavors.

The purpose of postoperative adjuvant therapy is to target tumor cells released during surgery, as well as microscopic lesions that may go undetected by imaging techniques [48]. Patients with HCC who have high-risk recurrence factors often present with more invasive primary lesions, increasing the possibility of residual tumor. Our findings indicated that ICIs&TKIs (SUCRA = 87.33%) was the best regimen for OS and performs significantly better than HAIC (SUCRA = 8.02%) and surgery alone (SUCRA = 6.47%), with clinical significance. We also found that ICIs&TKIs have shown an overwhelming advantage in improving the survival prognosis of patients with high-risk recurrence factors when compared with adjuvant TACE, RT and Aspirin. A single-arm study has demonstrated that combination of TKIs and anti-PD-1 antibodies achieved a surgical conversion rate of 55.4% in advanced HCC, with a median OS of 23.9 months [49]. For HCC patients with high-risk recurrent factors, ICIs&TKIs can increase the 3-year OS rate by 36.7% [32]. The above research findings highlighted its potent antitumor efficacy. This may be attributed to the synergistic anti-tumor effects of the combined regimens, which is more beneficial for clearing the residual micrometastatic lesions in the remaining liver, thus achieving satisfactory effects of prolonging long-term survival. Furthermore, we also found that the use of ICIs alone (SUCRA = 85.82%) has also shown excellent efficacy, suggesting their potential as a valuable standalone option in postoperative adjuvant therapy following liver resection. Regarding RFS, we found that TKIs&TACE was the best regimen (SUCRA value 84.08%), with a significantly higher SUCRA value compared to RT (75.32%), ICIs&TKIs (69.34%), ICIs (64.55%), HAIC (43.17%), TACE (33.97%), TKIs (28.39%) and surgery alone (1.19%). Great efforts have been made to explore effective preventive interventions to reduce the HCC recurrence after hepatectomy, among which TACE and Lenvatinib were two widely used therapies [50]. TACE, as a local treatment, mainly affected the blood vessels of the microvascular invasion lesions in the surgical area, while the systemic treatment of Lenvatinib mainly inhibited tumor cell proliferation and angiogenesis. Of note, TACE was often powerless for tumor cells shed by intraoperative manipulation and residual tumor cells in the cut edge or blood vessels. Therefore, the combination of the two may show the effect of “1 + 1>2” [51].

Postoperative TACE treatment is the most commonly used adjuvant treatment following surgery. However, there is ongoing debate regarding its effectiveness in reducing recurrence and prolonging survival in HCC patients [52]. Postoperative TACE may induce a hypoxic environment that stimulates the expression of hypoxia-inducible factor-1α and vascular endothelial growth factor (VEGF), activating related signaling pathways, promoting angiogenesis, and fostering a microenvironment that supports tumor growth, potentially leading to recurrence, progression, and metastasis [53,54]. Some studies have reported that postoperative TACE showed no significant benefit for HCC patients [55], while others indicated that it may achieve higher OS and RFS rates compared to simple surgical resection, supporting its role as an effective adjuvant treatment following hepatectomy [29,43]. For patients at high risk of recurrence, the direct antitumor effects of postoperative TACE appeared to outweigh its potential tumor-promoting effects, demonstrating considerable efficacy. However, in patients without MVI, with tumors≤5 cm in diameter, or with preoperative alpha-fetoprotein (AFP) levels<400 ng/ml, TACE may not improve long-term survival rates and could even exacerbate the risk of postoperative recurrence [56,57]. Our study revealed that TACE monotherapy has no significant advantage in prolonging OS or RFS for HCC patients with high-risk recurrence factors. However, indirect comparisons suggested a potential OS and RFS benefit with TKIs&TACE versus monotherapies, though this could not be verified through direct head-to-head trials due to limited evidence connections in the network.

Traditionally, RT has been avoided in the treatment of HCC due to concerns about the risk of radiation-induced liver disease and its limited efficacy [58]. However, advancements in technology have led to the development of sophisticated external RT techniques, such as three-dimensional conformal RT and intensity-modulated RT, which allowed for precise delivery of tumoricidal radiation dose to the tumor bed while sparing significant amounts of normal liver tissue [59]. Recent studies have also indicated that RT can remodel the tumor immune microenvironment through various mechanisms, including the modulation of exosomes and stromal cells, thereby enhancing its antitumor effects [60,61]. A series of studies have shown that RT increased effectiveness in HCC patients across all stages [62–65]. For patients with high-risk recurrence factors such as narrow or positive surgical margins [10,35], portal vein invasion [21], and MVI [28], postoperative RT has been shown to improve the prognosis. One study that compared the adjuvant treatment outcomes of RT versus TACE in HCC patients with positive MVI found that RT significantly outperformed TACE in terms of both OS and RFS [44]. Our network meta-analysis suggests that, compared to TACE, RT can prolong RFS in patients with high-risk recurrence factors, but no significant difference was observed in OS. Therefore, a comprehensive assessment of the relative advantages and disadvantages of RT compared to other postoperative adjuvant treatment strategies should rely on real-world studies with larger sample sizes.

TKIs such as sorafenib and lenvatinib were initially developed for the treatment of advanced HCC. Their mechanisms of action involved direct inhibition of tumor growth by targeting the RAF/MEK/ERK signaling pathway, as well as indirect inhibition of tumor cell proliferation by blocking vascular endothelial growth factor receptor (VEGFR) and platelet-derived growth factor receptor (PDGFR) pathways, which were crucial for tumor neovascularization [66]. Multiple RCTs have demonstrated that both sorafenib and lenvatinib significantly prolong OS and progression-free survival (PFS) in advanced HCC [67,68]. In terms of postoperative adjuvant therapy, multiple retrospective studies and small clinical trials have indicated that sorafenib as an adjuvant treatment can reduce recurrence risk and enhance long-term prognosis [31,69]. Nevertheless, the STORM trial, a rigorously designed randomized clinical trial, failed to provide conclusive evidence for the definitive efficacy of sorafenib as an adjuvant therapy [70]. In contrast, ICIs present distinct advantages as an adjuvant strategy following hepatectomy. ICIs not only reduced the risk of early recurrence by targeting hidden residual lesions but also activated the immune system to lower the incidence of new HCC cases [71,72]. Our network mete-analysis showed that postoperative administration of TKIs, ICIs, or combination therapy enhances both OS and RFS in liver cancer patients with high-risk recurrence factors compared to surgery alone.

Unlike systemic chemotherapy, HAIC can directly deliver drugs to the tumor-feeding artery to increase the local concentration, thereby achieving better inhibition of tumor recurrence and milder adverse reactions [73]. Numerous studies have observed that HAIC can reduce recurrence risk after hepatectomy in HCC patients with macroscopic PVTT, suggesting that patients with high-grade vascular invasion may be particularly suitable candidates for adjuvant HAIC [74,75]. A meta-analysis indicated that adjuvant HAIC improves PFS and OS after hepatectomy, especially for tumors larger than 7 cm [76]. A recent RCT demonstrated that while HAIC improved DFS in HCC patients with MVI, it did not significantly impact OS [11]. Moreover, a multicenter, open-label RCT [77] demonstrated that FOLFOX-HAIC significantly improved OS versus TACE in unresectable large HCC (tumor diameter≥7 cm). Our network meta-analysis indicates that, for HCC patients with high-risk recurrence factors, HAIC does not significantly improve OS or RFS. This lack of impact may be attributable to the broad range of identified high-risk factors and associated heterogeneity. Assessing HAIC’s efficacy for these patients would require more real-world data.

This study has several limitations. First, the current literature for network meta-analysis includes prospective and retrospective cohort studies as well as RCTs, which are susceptible to various biases. Second, the stringent inclusion criteria resulted in a limited number of eligible studies for this meta-analysis. Notably, certain intervention comparisons (e.g., ICIs&TKIs vs. surgery, TKIs&TACE vs. TACE) were informed by single-study evidence, which may compromise the robustness of the corresponding conclusions. Third, most sources for this study are from mainland China, with one from Taiwan. Given the differing etiologies of HCC in China versus Western countries, these results may not be globally generalizable. Last, this study includes only TKIs and ICIs that are currently widely used in clinical practice; the potential impact of newer adjuvant drugs on patient prognosis remains an area of interest. Therefore, this study should not be considered definitive.

In conclusion, our network meta-analysis indicates that, for HCC patients with high-risk recurrence factors, multiple adjuvant treatments such as ICIs, TKIs, RT, TACE, ICIs&TKIs, and TKIs&TACE can improve OS and RFS compared to surgery alone. For OS, ICIs&TKIs demonstrated superior efficacy to surgery alone and adjuvant HAIC. For RFS, TKIs&TACE significantly prolonged RFS compared to surgery alone and adjuvant HAIC.

## Supporting information

S1 File Search strategy. (ZIP)

S2 File PRISMA (Preferred Reporting Items for Systematic Reviews and Meta-Analyses) flow diagram. (ZIP)

S3 File Baseline characteristics. (ZIP)

S4 File GRADE assessment of certainty of evidence in RCT studies. (ZIP)

S5 File RoB 2 tool for Assessing risk of bias in RCT studies. (ZIP)

S6 File ROBINS-I tool for assessing risk of bias in non-randomized studies. (ZIP)

S7 File Trace and density plot of different interventions (OS). TACE, transhepatic arterial chemoembolization; HAIC, hepatic artery infusion chemotherapy; RT, radiotherapy; ICIs, immune checkpoint inhibitors; TKIs, Tyrosine kinase inhibitors; ICIs&TKIs, ICIs combined with TKIs; TKIs&TACE, TKIs combined with TACE.
(ZIP)

S8 File Trace and density plot of different interventions (RFS). TACE, transhepatic arterial chemoembolization; HAIC, hepatic artery infusion chemotherapy; RT, radiotherapy; ICIs, immune checkpoint inhibitors; TKIs, Tyrosine kinase inhibitors; ICIs&TKIs, ICIs combined with TKIs; TKIs&TACE, TKIs combined with TACE.
(ZIP)

S9 File Brooks-Gelman-Rubin diagnosis plot diagram of different interventions (OS). TACE, transhepatic arterial chemoembolization; HAIC, hepatic artery infusion chemotherapy; RT, radiotherapy; ICIs, immune checkpoint inhibitors; TKIs, Tyrosine kinase inhibitors; ICIs&TKIs, ICIs combined with TKIs; TKIs&TACE, TKIs combined with TACE.
(ZIP)

S10 File Brooks-Gelman-Rubin diagnosis plot diagram of different interventions (RFS). TACE, transhepatic arterial chemoembolization; HAIC, hepatic artery infusion chemotherapy; RT, radiotherapy; ICIs, immune checkpoint inhibitors; TKIs, Tyrosine kinase inhibitors; ICIs&TKIs, ICIs combined with TKIs; TKIs&TACE, TKIs combined with TACE.
(ZIP)

S11 File Heterogeneity test of different interventions (OS). TACE, transhepatic arterial chemoembolization; HAIC, hepatic artery infusion chemotherapy; RT, radiotherapy; ICIs, immune checkpoint inhibitors; TKIs, Tyrosine kinase inhibitors; ICIs&TKIs, ICIs combined with TKIs; TKIs&TACE, TKIs combined with TACE.
(ZIP)

S12 File Heterogeneity test of different interventions (RFS). TACE, transhepatic arterial chemoembolization; HAIC, hepatic artery infusion chemotherapy; RT, radiotherapy; ICIs, immune checkpoint inhibitors; TKIs, Tyrosine kinase inhibitors; ICIs&TKIs, ICIs combined with TKIs; TKIs&TACE, TKIs combined with TACE.
(ZIP)

S13 File Consistency test of different interventions (OS). TACE, transhepatic arterial chemoembolization; RT, radiotherapy.
(ZIP)

S14 File Consistency test of different interventions (RFS). TACE, transhepatic arterial chemoembolization; RT, radiotherapy; TKIs, Tyrosine kinase inhibitors; TKIs&TACE, TKIs combined with TACE.
(ZIP)

S15 File Funnel plot for the efficacy of adjuvant treatments on contributing OS (A) and preventing recurrence (B). (ZIP)

S16 File Prisma statements 2020. (ZIP)

S1 Data The raw data of this study. (ZIP)

S2 Data The included and excluded articles of this study. (ZIP)
