# Supplementary material for: Comparative effectiveness of adjuvant treatment for hepatocellular carcinoma with high risk of recurrence: A systematic review and network meta-analysis
Source: PLoS One. 2025 Dec 4;20(12):e0335457. doi: 10.1371/journal.pone.0335457 (PMC12677550; doi:10.1371/journal.pone.0335457)
Supplement: S1 File — (ZIP) [file pone.0335457.s001.zip › Supplementary Material/S1 File.docx]

| **Search number** | **Search term** | **Citations** |
| --- | --- | --- |
| Medline |  |  |
|  | **((Adjuvant[Title/Abstract]) OR (postoperative[Title/Abstract])) AND (((Curative resection[Title/Abstract]) OR (R0 resection[Title/Abstract])) AND ((("liver cancer"[Title/Abstract]) OR ("Hepatocellular Carcinoma"[Title/Abstract]) OR ("Liver Neoplasms"[Mesh] )OR ("Carcinoma, Hepatocellular"[Mesh]) OR (HCC[Title/Abstract])) AND ((liver resection[Title/Abstract]) OR (Hepatectomy[Title/Abstract]) OR (hepatic resection[Title/Abstract])OR (hepatectomy[MeSH Terms]))))** | 701 |
| Embase |  |  |
|  | **#13. #7 AND #10 AND #11 AND #12** | **1,061** |
|  | **#12. adjuvant:ab,ti OR postoperative:ab,ti** | **1,196,537** |
|  | **#11. #8 OR #9** | **83,668** |
|  | **#10. 'curative resection':ab,ti OR 'r0 resection':ab,ti** | **30,314** |
|  | **#9. 'hepatectomy'/exp** | **75,880** |
|  | **#8. 'liver resection':ab,ti OR hepatectomy:ab,ti OR**  **'hepatic resection':ab,ti** | **58,562** |
|  | **#7. #1 OR #2 OR #3 OR #4 OR #5 OR #6** | **385,544** |
|  | **#6. 'liver cell carcinoma'/exp** | **226,197** |
|  | **#5. 'liver cancer'/exp** | **348,089** |
|  | **#4. 'hcc':ab,ti** | **129,362** |
|  | **#3. 'liver neoplasms':ab,ti** | **733** |
|  | **#2. 'hepatocellular carcinoma':ab,ti** | **177,727** |
|  | **#1. 'liver cancer':ab,ti** | **43,497** |
| Cochrane Central Register of Controlled Trials |  |  |
|  | **#1 (liver resection):ti,ab,kw OR (Hepatectomy):ti,ab,kw OR (hepatic resection):ti,ab,kw (Word variations have been searched)** | **6154** |
|  | **#2 MeSH descriptor: [Liver Neoplasms] explode all trees** | **4506** |
|  | **#3 (Adjuvant):ti,ab,kw OR (postoperative):ti,ab,kw (Word variations have been searched)** | **208891** |
|  | **#4 ("curative resection"):ti,ab,kw OR (R0 resection):ti,ab,kw (Word variations have been searched)** | **3657** |
|  | **#5 #1 OR #2** | **9292** |
|  | **#6 #5 AND #3 AND #4** | 531 |
|  | **#7 ("liver cancer"):ti,ab,kw OR ("Hepatocellular Carcinoma"):ti,ab,kw OR ("Liver Neoplasms"):ti,ab,kw (Word variations have been searched)** | **8965** |
|  | **#8 MeSH descriptor: [Liver Neoplasms] explode all trees** | **4506** |
|  | **#9 #7 OR #8** | **9105** |
|  | **#10 #6 AND #9** | 243 |
| **Web of science** |  |  |
|  | **#1 TS=("liver cancer" OR "hepatocellular carcinoma" OR "'liver neoplasms" OR HCC OR "liver tumor")** | **391,209** |
|  | **#2 TS=("curative resection" OR "R0 resection")** | **25066** |
|  | **#3 TS=(Adjuvant OR postoperative)** | **1,397,468** |
|  | **#4 TS=("liver resection" OR "hepatic resection" OR Hepatectomy)** | **67,722** |
|  | **#1 AND #2 AND #3 AND #4 A** | **1246** |
