# Supplementary material for: Comparative effectiveness of adjuvant treatment for hepatocellular carcinoma with high risk of recurrence: A systematic review and network meta-analysis
Source: PLoS One. 2025 Dec 4;20(12):e0335457. doi: 10.1371/journal.pone.0335457 (PMC12677550; doi:10.1371/journal.pone.0335457)
Supplement: S1 File — (ZIP) [file pone.0335457.s001.zip › Supplementary Material/S3 File.docx]

| Supplementary Table 2 Basic characteristics of the included studies | | | | | | | | | | | | | | | | | |
| --- | --- | --- | --- | --- | --- | --- | --- | --- | --- | --- | --- | --- | --- | --- | --- | --- | --- |
| Study | year | PMID | Study type | Begin and end of Study | Crowd characteristics | Treatment | Sample  size(n) | Gender, M/F  (n) | Age (year) | Number of tumors,  S/M (n) | Tumor size  (cm) | Liver cirrhosis (n) | Virology, HBV/HCV (n) | MVI (n) | Edmondson’s grading, I–II/III–IV,(n) | Resection margin(＜1cm or positive), n | Main outcomes |
| Xiang(27) | 2024 | 37812183 | Prospective study | 2013.1-2019.12 | Intermediate-stage HCC with MVI | TACE | 90.00 | 79/11 | 53 [18-79] | 0/90 | 7.0 [5.0-19.3] | 65 | NR | 90 | 64/26 | 80 | OS，RFS |
|  |  |  |  |  |  | Hepatectomy  alone | 90.00 | 77/13 | 53 [19-80] | 0/90 | 7.0 [4.9-18.0] | 64 | NR | 90 | 70/20 | 79 | OS，RFS |
| Peng(26) | 2024 | 38568599 | RCT | 2019.10-2022.3 | HCC with Cheng grade I to III PVTT | Sorafenib+  TACE | 79.00 | 69/10 | 55(47-64) | 37/42 | 6.3(4.5-9.5) | 53 | 71/4 | NR | NR | NR | OS，RFS |
|  |  |  |  |  |  | Sorafenib | 79.00 | 71/8 | 52(42-61 | 33/46 | 7.5(5.0-10.0) | 53 | 72/2 | NR | NR | NR | OS，RFS |
| Bai(2) | 2024 | 38488934 | RCT | 2017.8-2019.7 | hepatectomy with narrow  pathologic margins (< 1 cm) | RT | 24.00 | 23/2 | 47.3 ± 10.8 | 22/2 | NR | 9 | 18/1 | 16 | 15/9 | 24 | OS，RFS |
|  |  |  |  |  |  | TACE | 48.00 | 41/7 | 49.0 ± 11.6 | 38/10 | NR | 25 | 43/2 | 22 | 33/15 | 48 | OS，RFS |
| Wang(32) | 2024 | 38242982 | RCT | 2020.9-2022.4 | HCC with MVI | Sintilimab | 99.00 | 85/14 | 53.0 (48.0–61.0) | 87/12 | NR | 44 | 70/3 | 99 | 54/45 | NR | OS，RFS |
|  |  |  |  |  |  | Hepatectomy alone | 99.00 | 83/16 | 54.0 (49.0–61.0) | 86/13 | NR | 56 | 75/2 | 99 | 58/41 | NR | OS，RFS |
| Lu(19) | 2023 | 37810112 | RCT | 2013.6-2015.7 | HCC with PVTT | Aspirin | 40.00 | 37/3 | NR | 37/3 | NR | 7 | NR | 36 | NR | NR | OS |
|  |  |  |  |  |  | Hepatectomy alone | 40.00 | 37/3 | NR | 38/2 | NR | 8 | NR | 37 | NR | NR | OS |
| Luo(47) | 2023 | 36905230 | Retrospective study | 2018.1-2021.9 | HCC with MVI | TACE | 266.00 | NR | NR | NR | NR | NR | NR | NR | NR | NR | OS，DFS |
|  |  |  |  |  |  | Hepatectomy alone | 249.00 | NR | NR | NR | NR | NR | NR | NR | NR | NR | OS，DFS |
| Long(10) | 2023 | 36634853 | Prospective study | 2008.1-2016.3 | hepatectomy with narrow  pathologic margins (< 1 cm) | RT | 65.00 | 59/6 | 52(31–75 | 63/2 | NR | NR | NR | 6 | NR | NR | OS，DFS |
|  |  |  |  |  |  | Hepatectomy alone | 65.00 | 56/9 | 53(27–80) | 63/2 | NR | NR | NR | 11 | NR | NR | OS，DFS |
| Li(12) | 2023 | 37452107 | Prospective study | 2019.3-2022.3 | HCC with high risk of recurrence | ICIs alone or with TKIs | 74.00 | 66/8 | 50.6±9.6 | 53/21 | 6.7±4.2 | 57 | NR | 34 | NR | NR | OS，RFS |
|  |  |  |  |  |  | Hepatectomy alone | 148.00 | 131/17 | 50.6±11.3 | 106/42 | 6.8±3.9 | 113 | NR | 69 | NR | NR | OS，RFS |
| Li(32) | 2023 | 37359534 | Retrospective study | 2019.1-2021.12 | HCC with High risk of recurrence | ICIs +TKIs | 47 | 43/4 | 35/12 | NR | NR | NR | NR | 33 | 14/33 | NR | OS，RFS |
|  |  |  |  |  |  | Hepatectomy alone | 47 | 42/5 | 34/13 | NR | NR | NR | NR | 32 | 13/34 | NR | OS，RFS |
| Bai(33) | 2023 | 37029989 | Retrospective study | 2016.1-2017.12 | HCC with narrow margin | TACE | 70 | 61/9 | NR | NR | NR | 52 | NR | 43 | 17/53 | 70 | OS，RFS |
|  |  |  |  |  |  | Hepatectomy  alone | 70 | 58/12 | NR | NR | NR | 51 | NR | 39 | 16/54 | 70 | OS，RFS |
| Li(11) | 2023 | 36525610 | RCT | 2016.6-2021.8 | HCC with MVI | HAIC | 157 | 136/21 | 50(25-75 | 114/43 | 5.6(1.8-30.0) | NR | 137/11 | 157 | 63/92 | NR | OS，DFS |
|  |  |  |  |  |  | Hepatectomy  alone | 158 | 139/19 | 54(27-75) | 128/30 | 5.4(1.5-16.0) | NR | 138/6 | 158 | 78/80 | NR | OS，DFS |
| Gou(35) | 2022 | 35643251 | Retrospective study | 2011.1-2020.12 | HCC with narrow or  positive margins | RT | 78 | 67/11 | 52 ± 10 | 61/17 | NR | 53 | NR | 33 | NR | NR | OS，RFS |
|  |  |  |  |  |  | Hepatectomy alone | 78 | 70/8 | 51 ± 11 | 65/13 | NR | 52 | NR | 34 | NR | NR | OS，RFS |
| Lin(34) | 2022 | 35300207 | Retrospective study | 2014.2-2021.1 | HCC with high risk of recurrence | TACE+TKIs | 48 | 39/9 | 50.0 ± 12.3 | NR | 9.17±4.13 | 34 | NR | 46 | NR | NR | DFS |
|  |  |  |  |  |  | TACE | 48 | 39/9 | 51.3 ± 12.0 | NR | 8.55± 3.99 | 33 | NR | 45 | NR | NR | DFS |
| Qiu(13) | 2022 | 35795039 | Retrospective study | 2014.4-2019.7 | HCC with MVI | TACE | 164 | 138/26 | 51±12 | 121/43 | NR | 86 | NR | 165 | 65/99 | NR | OS，RFS |
|  |  |  |  |  |  | Hepatectomy alone | 164 | 145/19 | 52±12 | 112/52 | NR | 78 | NR | 165 | 66/98 | NR | OS，RFS |
| Wang(37) | 2021 | 33455865 | Retrospective study | 2009.12-2010.12 | HCC diameter ≥10 cm | TACE | 69 | 59/10 | 50.39 ±9.79 | 51/18 | 13.48 ±2.99 | 27 | NR | 45 | 7/62 | NR | OS，RFS |
|  |  |  |  |  |  | Hepatectomy alone | 69 | 60/9 | 51.07 ±11.68 | 50/19 | 13.18 ±2.93 | 24 | NR | 45 | 9/60 | NR | OS，RFS |
| Li(38) | 2021 | 34631511 | Retrospective study | 2009.8-2017.8 | HCC with MVI | Sorafenib | 42 | 34/8 | 54.2±1.4 | 13/29 | 6.2±0.6 | 34 | NR | 42 | 7/63 | NR | OS，RFS |
|  |  |  |  |  |  | Hepatectomy alone | 42 | 35/7 | 54.6±1.7 | 17/25 | 7.2±0.8 | 37 | NR | 42 | 9/61 | NR | OS，RFS |
| Wang(36) | 2021 | 32440804 | Retrospective study | 2012.12-2015.12 | Intermediate HCC | TACE | 123 | 108/15 | NR | 0/123 | NR | 85 | NR | 55 | 120/3 | NR | OS，DFS |
|  |  |  |  |  |  | Hepatectomy alone | 123 | 111/12 | NR | 0/123 | NR | 85 | NR | 59 | 119/4 | NR | OS，DFS |
| Huang(40) | 2020 | 33061610 | Retrospective study | 2008.1-2017.12 | HCC with macroscopic bile duct tumor thrombus | TACE | 31 | 28/3 | 51.0 (45.5, 59.0) | 24/7 | NR | 24 | NR | 26 | 7/24 | NR | OS，RFS |
|  |  |  |  |  |  | Hepatectomy alone | 31 | 27/4 | 54.0(48.0, 59.0) | 27/4 | NR | 29 | NR | 26 | 3/28 | NR | OS，RFS |
|  |  |  |  |  |  |  |  |  |  |  |  |  |  |  |  |  |  |
| Rong(20) | 2020 | 33223759 | RCT | 2007.7-2012.3 | HCC with narrow-margin | RT | 58 | 51/7 | 53.1±10.5 | NR | 4.7± 2.6 | 51 | NR | NR | NR | 58 | OS，RFS |
|  |  |  |  |  |  | Hepatectomy alone | 61 | 48/13 | 55.5 ±10.7 | NR | 5.6 ± 3.7 | 54 | NR | NR | NR | 61 | OS，RFS |
| Wang(28) | 2020 | 32611327 | Prospective study | 2015.7-2018.12 | HCC with MVI | RT | 29 | 24/5 | 55.90±8.05 | 27/2 | 4.75±2.15 | NR | NR | 29 | NR | NR | OS，RFS |
|  |  |  |  |  |  | Hepatectomy alone | 30 | 25/5 | 56.57±9.43 | 28/2 | 4.50±2.98 | NR | NR | 30 | NR | NR | OS，RFS |
| Wang(39) | 2020 | 32547217 | Retrospective study | 2013.12-2015.12 | HCC with MVI | TACE | 199 | 176/23 | NR | 63/136 | NR | 143 | NR | 199 | NR | NR | OS，DFS |
|  |  |  |  |  |  | Hepatectomy alone | 199 | 173/26 | NR | 61/138 | NR | 144 | NR | 199 | NR | NR | OS，DFS |
| Zhang(41) | 2019 | 30767178 | Retrospective study | 2002.1-2015.12 | HCC with hepatic vein tumor thrombus | TACE | 107 | 90/17 | 54.2±10.17 | 79/28 | 10.0 (7.0-12.0) | NR | NR | NR | NR | NR | OS，RFS |
|  |  |  |  |  |  | Hepatectomy alone | 107 | 91/16 | 54.7±10.69 | 74/33 | 10.3 (8.7-12.0) | NR | NR | NR | NR | NR | OS，RFS |
| Zhang(42) | 2019 | 31153833 | Retrospective study | 2009-2016 | HCC with MVI | Sorafenib | 113 | 97/16 | 49.0(43.0–56.0 | 96/17 | 5.9(4.0–9.0 | 69 | NR | 113 | 12/101 | NR | OS，RFS |
|  |  |  |  |  |  | Hepatectomy alone | 113 | 98/15 | 48.0(40.0–57.0 | 92/21 | 5.42(3.8–9.1) | 82 | NR | 113 | 19/94 | NR | OS，RFS |
| Wang(44) | 2019 | 30863091 | Retrospective study | 2008.7-2016.12 | HCC with MVI | RT | 46 | 43/3 | 50.98±10.53 | 42/4 | 5.39±2.74 | 41 | NR | 46 | NR | NR | OS，RFS |
|  |  |  |  |  |  | TACE | 46 | 37/9 | 51.52±11.40 | 41/5 | 5.50±3.07 | 39 | NR | 46 | NR | NR | OS，RFS |
| Sun(21) | 2019 | 31176205 | RCT | 2013.7-2016.6 | HCC with PVTT | RT | 26 | 24/2 | 49.6±7.7 | 25/1 | NR | NR | NR | NR | NR | NR | OS，DFS |
|  |  |  |  |  |  | Hepatectomy alone | 26 | 24/2 | 51.1±10.8 | 24/2 | NR | NR | NR | NR | NR | NR | OS，DFS |
| Qi(29) | 2019 | 30103903 | Prospective study | 2012.1-2014.12 | HCC with MVI | TACE | 91 | 78/13 | NR | 68/23 | NR | 79 | NR | 91 | NR | NR | OS，DFS |
|  |  |  |  |  |  | Hepatectomy alone | 109 | 93/16 | NR | 84/25 | NR | 89 | NR | 109 | NR | NR | OS，DFS |
| Wang(43) | 2019 | 30249510 | Retrospective study | 2004.9-2015.12 | HCC with MVI | TACE | 57 | 47/10 | 55±11 | 46/11 | 6(2-14) | 49 | NR | 57 | 41/16 | NR | OS，RFS |
|  |  |  |  |  |  | Hepatectomy alone | 57 | 51/6 | 56±10 | 46/11 | 6(2-18) | 46 | NR | 57 | 41/16 | NR | OS，RFS |
| Wei(22) | 2018 | 30305149 | RCT | 2009.6-2012.12 | HCC with a solitary tumor ≥ 5 cm and MVI | TACE | 116 | 106/10 | 44.0 (18–75) | 116/0 | NR | 50 | NR | 116 | NR | NR | OS，DFS |
|  |  |  |  |  |  | Hepatectomy alone | 118 | 106/12 | 48.5 (18–74) | 118/0 | NR | 42 | NR | 118 | NR | NR | OS，DFS |
| Wang(23) | 2018 | 29420221 | RCT | 2011.8-2014.8 | HCC intermediate or high  risk recurrence factors | TACE | 140 | 121/19 | 54.2±9.7 | 102/38 | NR | NR | NR | 78 | 81/59 | NR | OS，RFS |
|  |  |  |  |  |  | Hepatectomy alone | 140 | 109/31 | 52.6±10.3 | 109/31 | NR | NR | NR | 87 | 80/60 | NR | OS，RFS |
| Li(45) | 2017 | 28032575 | Retrospective study | 2007-2013 | HCC beyond the Milan criteria | TACE | 284 | 53/231 | 50.6± 11.1 | 223/61 | 8.2 ± 3.3 | 256 | 279/5 | 126 | NR | NR | OS，RFS |
|  |  |  |  |  |  | Hepatectomy alone | 284 | 48/236 | 50.9 ± 12.3 | 221/63 | 8.2 ± 3.0 | 255 | 279/5 | 117 | NR | NR | OS，RFS |
| Hsiao(30) | 2017 | 28728985 | Prospective study | 2006-2014 | HCC≥T2 tumors (7th AJCC TNM staging system) | HAIC | 61 | 51/10 | 58.0(24-77) | 28/33 | NR | 23 | NR | NR | NR | NR | OS，DFS |
|  |  |  |  |  |  | Hepatectomy alone | 160 | 126/34 | 63.3(27-86) | 89/71 | NR | 63 | NR | NR | NR | NR | OS，DFS |
| Xia(31) | 2016 | 27340354 | Prospective study | 2010.9-2013.9 | BCLC-stage C HCC | Sorafenib | 34 | 25/9 | 48 (21-78) | NR | 6.4 (2.8-20.2) | 30 | NR | NR | NR | NR | OS，DFS |
|  |  |  |  |  |  | Hepatectomy alone | 68 | 50/18 | 57 (18-79) | NR | 5.9 (2.9-21.3) | 60 | NR | NR | NR | NR | OS，DFS |
| Li(46) | 2015 | 24972992 | Retrospective study | 2006.2-2009.5 | BCLC-stage B HCC | TACE | 26 | 22/4 | 53.59±10.19 | 22/4 | 4.89±2.81 | 17 | NR | NR | NR | NR | OS |
|  |  |  |  |  |  | Hepatectomy alone | 34 | 30/4 | 51.56±9.35 | 26/8 | 5.07±3.13 | 21 | NR | NR | NR | NR | OS |
| Zhong(24) | 2009 | 19408012 | RCT | 2001.1-2004.3 | Stage IIIA HCC | TACE | 57 | 53/4 | 47.6±10.4 | 0/57 | 9.5±3.8 | NR | NR | NR | 35/22 | NR | OS，DFS |
|  |  |  |  |  |  | Hepatectomy alone | 58 | 49/9 | 48.2±11.2 | 0/58 | 9.7±3.6 | NR | NR | NR | 34/24 | NR | OS，DFS |
| Peng(25) | 2009 | 19285298 | RCT | 1996.1-2004.12 | HCC with PVTT | TACE | 51 | 46/5 | 46.2 ±13.8 | NR | 90.4 ±30.2 | 42 | 31/5 | NR | NR | NR | OS |
|  |  |  |  |  |  | Hepatectomy alone | 53 | 50/3 | 50.2 ±7.5 | NR | 83.9 ±22. | 37 | 40/3 | NR | NR | NR | OS |

S, single; M, multiple; OS, overall survival; DFS, disease-free survival; RFS，recurrence-free survival; RCT, randomized controlled trial; TACE, transhepatic arterial chemoembolization; HAIC, hepatic artery infusion chemotherapy; RT, radiotherapy; ICIs, immune checkpoint inhibitors; TKIs, Tyrosine kinase inhibitors; ICIs&TKIs, ICIs combined with TKIs; TKIs&TACE, TKIs combined with TACE; HBV, Hepatitis B virus; HCV, Hepatitis C virus; MVI, microvascular invasion; PVTT, portal vein tumor thrombosis; NR, not reported.
