# Supplementary material for: Comparative effectiveness of adjuvant treatment for hepatocellular carcinoma with high risk of recurrence: A systematic review and network meta-analysis
Source: PLoS One. 2025 Dec 4;20(12):e0335457. doi: 10.1371/journal.pone.0335457 (PMC12677550; doi:10.1371/journal.pone.0335457)
Supplement: S1 File — (ZIP) [file pone.0335457.s001.zip › Supplementary Material/S4 File.docx]

| **Sorafenib+TACE compared to Sorafenib for HCC with Cheng grade I to III PVTT** | | | | | | |
| --- | --- | --- | --- | --- | --- | --- |
| **Patient or population:** patients with HCC with Cheng grade I to III PVTT **Settings:** multicenter **Intervention:** Sorafenib+TACE  **Comparison:** Sorafenib | | | | | | |
| **Outcomes** | **Illustrative comparative risks* (95% CI)** | | **Relative effect (95% CI)** | **No of Participants (studies)** | **Quality of the evidence (GRADE)** | **Comments** |
|  | Assumed risk | Corresponding risk |  |  |  |  |
|  | **Sorafenib** | **Sorafenib+TACE** |  |  |  |  |
| **Overall survival** Follow-up: mean 28.4 months | **Study population** | | **HR 0.57**  (0.36 to 0.91) | 158 (1 study) | ⊕⊕⊕⊝ **moderate**^2^ |  |
|  | See comment | See comment |  |  |  |  |
|  | **Moderate** | |  |  |  |  |
|  |  | ^1^ |  |  |  |  |
| **Recurrence free survival** Follow-up: mean 28.4 months | **Moderate** | | **HR 0.57**  (0.39 to 0.83) | 158 (1 study) | ⊕⊕⊕⊝ **moderate**^2^ |  |
|  |  |  |  |  |  |  |
| *The basis for the **assumed risk** (e.g. the median control group risk across studies) is provided in footnotes. The **corresponding risk** (and its 95% confidence interval) is based on the assumed risk in the comparison group and the **relative effect** of the intervention (and its 95% CI).  **CI:** Confidence interval; **HR:** Hazard ratio; | | | | | | |
| GRADE Working Group grades of evidence **High quality:** Further research is very unlikely to change our confidence in the estimate of effect.  **Moderate quality:** Further research is likely to have an important impact on our confidence in the estimate of effect and may change the estimate. **Low quality:** Further research is very likely to have an important impact on our confidence in the estimate of effect and is likely to change the estimate. **Very low quality:** We are very uncertain about the estimate. | | | | | | |
| ^1^ No explanation was provided ^2^ Blinding was not implemented | | | | | | |

|  | | | | | | |
| --- | --- | --- | --- | --- | --- | --- |
| **Patient or population:** patients with hepatectomy with narrow pathologic margins  **Settings:**  **Intervention:** RT  **Comparison:** TACE | | | | | | |
| **Outcomes** | **Illustrative comparative risks* (95% CI)** | | **Relative effect (95% CI)** | **No of Participants (studies)** | **Quality of the evidence (GRADE)** | **Comments** |
|  | Assumed risk | Corresponding risk |  |  |  |  |
|  | **TACE** | **RT** |  |  |  |  |
| **OS** Follow-up: mean 44.4 months | **Study population** | | **HR 0.73**  (0.22 to 2.37) | 72 (1 study) | ⊕⊕⊕⊝ **moderate**^1^ |  |
|  | See comment | See comment |  |  |  |  |
|  | **Moderate** | |  |  |  |  |
|  | **750 per 1000** | **637 per 1000** (263 to 963) |  |  |  |  |
| **RFS** Follow-up: mean 44.4 | **Study population** | | **HR 0.78**  (0.3 to 2.0) | 72 (1 study) | ⊕⊕⊕⊝ **moderate**^1^ |  |
|  | See comment | See comment |  |  |  |  |
|  | **Moderate** | |  |  |  |  |
|  | **750 per 1000** | **661 per 1000** (340 to 938) |  |  |  |  |
| *The basis for the **assumed risk** (e.g. the median control group risk across studies) is provided in footnotes. The **corresponding risk** (and its 95% confidence interval) is based on the assumed risk in the comparison group and the **relative effect** of the intervention (and its 95% CI).  **CI:** Confidence interval; **HR:** Hazard ratio; | | | | | | |
| GRADE Working Group grades of evidence **High quality:** Further research is very unlikely to change our confidence in the estimate of effect.  **Moderate quality:** Further research is likely to have an important impact on our confidence in the estimate of effect and may change the estimate. **Low quality:** Further research is very likely to have an important impact on our confidence in the estimate of effect and is likely to change the estimate. **Very low quality:** We are very uncertain about the estimate. | | | | | | |
| ^1^ Blinding was not implemented | | | | | | |

| **Sintilimab compared to Hepatectomy alone for HCC with MVI** | | | | | | |
| --- | --- | --- | --- | --- | --- | --- |
| **Patient or population:** patients with HCC with MVI **Settings:**  **Intervention:** Sintilimab  **Comparison:** Hepatectomy alone | | | | | | |
| **Outcomes** | **Illustrative comparative risks* (95% CI)** | | **Relative effect (95% CI)** | **No of Participants (studies)** | **Quality of the evidence (GRADE)** | **Comments** |
|  | Assumed risk | Corresponding risk |  |  |  |  |
|  | **Hepatectomy alone** | **Sintilimab** |  |  |  |  |
| **OS**  Follow-up: mean 23.3 months | **Study population** | | **HR 0.505**  (0.254 to 1.006) | 198 (1 study) | ⊕⊕⊕⊝ **moderate**^1^ |  |
|  | See comment | See comment |  |  |  |  |
|  | **Moderate** | |  |  |  |  |
|  | **50 per 1000** | **26 per 1000** (13 to 50) |  |  |  |  |
| **RFS** Follow-up: mean 23.3 months | **Study population** | | **HR 0.534**  (0.360 to 0.792) | 198 (1 study) | ⊕⊕⊕⊝ **moderate**^1^ |  |
|  | See comment | See comment |  |  |  |  |
|  | **Moderate** | |  |  |  |  |
|  | **750 per 1000** | **523 per 1000** (393 to 666) |  |  |  |  |
| *The basis for the **assumed risk** (e.g. the median control group risk across studies) is provided in footnotes. The **corresponding risk** (and its 95% confidence interval) is based on the assumed risk in the comparison group and the **relative effect** of the intervention (and its 95% CI).  **CI:** Confidence interval; **HR:** Hazard ratio; | | | | | | |
| GRADE Working Group grades of evidence **High quality:** Further research is very unlikely to change our confidence in the estimate of effect.  **Moderate quality:** Further research is likely to have an important impact on our confidence in the estimate of effect and may change the estimate. **Low quality:** Further research is very likely to have an important impact on our confidence in the estimate of effect and is likely to change the estimate. **Very low quality:** We are very uncertain about the estimate. | | | | | | |
| ^1^ Blinding was not implemented | | | | | | |

| **Aspirin compared to Hepatectomy alone for HCC with PVTT** | | | | | | |
| --- | --- | --- | --- | --- | --- | --- |
| **Patient or population:** patients with HCC with PVTT **Settings:** the Eastern Hepatobiliary Surgery Hospital **Intervention:** Aspirin **Comparison:** Hepatectomy alone | | | | | | |
| **Outcomes** | **Illustrative comparative risks* (95% CI)** | | **Relative effect (95% CI)** | **No of Participants (studies)** | **Quality of the evidence (GRADE)** | **Comments** |
|  | Assumed risk | Corresponding risk |  |  |  |  |
|  | **Hepatectomy alone** | **Aspirin** |  |  |  |  |
| **OS** Follow-up: mean 19 months | **Study population** | | **HR 0.664**  (0.419 to 1.052) | 80 (1 study) | ⊕⊕⊕⊝ **moderate**^1^ |  |
|  | See comment | See comment |  |  |  |  |
|  | **Moderate** | |  |  |  |  |
|  | **750 per 1000** | **602 per 1000** (441 to 767) |  |  |  |  |
| *The basis for the **assumed risk** (e.g. the median control group risk across studies) is provided in footnotes. The **corresponding risk** (and its 95% confidence interval) is based on the assumed risk in the comparison group and the **relative effect** of the intervention (and its 95% CI).  **CI:** Confidence interval; **HR:** Hazard ratio; | | | | | | |
| GRADE Working Group grades of evidence **High quality:** Further research is very unlikely to change our confidence in the estimate of effect.  **Moderate quality:** Further research is likely to have an important impact on our confidence in the estimate of effect and may change the estimate. **Low quality:** Further research is very likely to have an important impact on our confidence in the estimate of effect and is likely to change the estimate. **Very low quality:** We are very uncertain about the estimate. | | | | | | |
| ^1^ Blinding was not implemented | | | | | | |

| **HAIC compared to Hepatectomy alone for HCC with MVI** | | | | | | |
| --- | --- | --- | --- | --- | --- | --- |
| **Patient or population:** patients with HCC with MVI **Settings:** multicenter trial **Intervention:** HAIC **Comparison:** Hepatectomy alone | | | | | | |
| **Outcomes** | **Illustrative comparative risks* (95% CI)** | | **Relative effect (95% CI)** | **No of Participants (studies)** | **Quality of the evidence (GRADE)** | **Comments** |
|  | Assumed risk | Corresponding risk |  |  |  |  |
|  | **Hepatectomy alone** | **HAIC** |  |  |  |  |
| **OS** Follow-up: mean 23.7 months | **Study population** | | **HR 0.64**  (0.36 to 1.14) | 315 (1 study) | ⊕⊕⊕⊝ **moderate**^1^ |  |
|  | See comment | See comment |  |  |  |  |
|  | **Moderate** | |  |  |  |  |
|  | **750 per 1000** | **588 per 1000** (393 to 794) |  |  |  |  |
| **DFS** Follow-up: mean 23.7 months | **Study population** | | **HR 0.59**  (0.43 to 0.81) | 315 (1 study) | ⊕⊕⊕⊝ **moderate**^1^ |  |
|  | See comment | See comment |  |  |  |  |
|  | **Moderate** | |  |  |  |  |
|  | **750 per 1000** | **559 per 1000** (449 to 675) |  |  |  |  |
| *The basis for the **assumed risk** (e.g. the median control group risk across studies) is provided in footnotes. The **corresponding risk** (and its 95% confidence interval) is based on the assumed risk in the comparison group and the **relative effect** of the intervention (and its 95% CI).  **CI:** Confidence interval; **HR:** Hazard ratio; | | | | | | |
| GRADE Working Group grades of evidence **High quality:** Further research is very unlikely to change our confidence in the estimate of effect.  **Moderate quality:** Further research is likely to have an important impact on our confidence in the estimate of effect and may change the estimate. **Low quality:** Further research is very likely to have an important impact on our confidence in the estimate of effect and is likely to change the estimate. **Very low quality:** We are very uncertain about the estimate. | | | | | | |
| ^1^ Blinding was not implemented | | | | | | |

| **RT compared to Hepatectomy alone for HCC with narrow-margin** | | | | | | |
| --- | --- | --- | --- | --- | --- | --- |
| **Patient or population:** patients with HCC with narrow-margin **Settings:** multicenter **Intervention:** RT  **Comparison:** Hepatectomy alone | | | | | | |
| **Outcomes** | **Illustrative comparative risks* (95% CI)** | | **Relative effect (95% CI)** | **No of Participants (studies)** | **Quality of the evidence (GRADE)** | **Comments** |
|  | Assumed risk | Corresponding risk |  |  |  |  |
|  | **Hepatectomy alone** | **RT** |  |  |  |  |
| **OS** Follow-up: mean 10 years | **Study population** | | **HR 0.85**  (0.48 to 1.50) | 119 (1 study) | ⊕⊕⊕⊝ **moderate**^1^ |  |
|  | See comment | See comment |  |  |  |  |
|  | **Moderate** | |  |  |  |  |
|  | **750 per 1000** | **692 per 1000** (486 to 875) |  |  |  |  |
| **RFS** Follow-up: mean 10 years | **Study population** | | **HR 0.62**  (0.33 to 1.14) | 119 (1 study) | ⊕⊕⊕⊝ **moderate**^1^ |  |
|  | See comment | See comment |  |  |  |  |
|  | **Moderate** | |  |  |  |  |
|  | **750 per 1000** | **577 per 1000** (367 to 794) |  |  |  |  |
| *The basis for the **assumed risk** (e.g. the median control group risk across studies) is provided in footnotes. The **corresponding risk** (and its 95% confidence interval) is based on the assumed risk in the comparison group and the **relative effect** of the intervention (and its 95% CI).  **CI:** Confidence interval; **HR:** Hazard ratio; | | | | | | |
| GRADE Working Group grades of evidence **High quality:** Further research is very unlikely to change our confidence in the estimate of effect.  **Moderate quality:** Further research is likely to have an important impact on our confidence in the estimate of effect and may change the estimate. **Low quality:** Further research is very likely to have an important impact on our confidence in the estimate of effect and is likely to change the estimate. **Very low quality:** We are very uncertain about the estimate. | | | | | | |
| ^1^ Blinding was not implemented | | | | | | |

| **RT compared to Hepatectomy alone for HCC with PVTT** | | | | | | |
| --- | --- | --- | --- | --- | --- | --- |
| **Patient or population:** patients with HCC with PVTT **Settings:** Eastern **Intervention:** RT  **Comparison:** Hepatectomy alone | | | | | | |
| **Outcomes** | **Illustrative comparative risks* (95% CI)** | | **Relative effect (95% CI)** | **No of Participants (studies)** | **Quality of the evidence (GRADE)** | **Comments** |
|  | Assumed risk | Corresponding risk |  |  |  |  |
|  | **Hepatectomy alone** | **RT** |  |  |  |  |
| **OS** Follow-up: mean 12 months | **Study population** | | **HR 0.444**  (0.243 to 0.813) | 52 (1 study) | ⊕⊕⊕⊝ **moderate**^1^ |  |
|  | See comment | See comment |  |  |  |  |
|  | **Moderate** | |  |  |  |  |
|  | **750 per 1000** | **460 per 1000** (286 to 676) |  |  |  |  |
| **DFS** Follow-up: mean 12 months | **Study population** | | **HR 0.358**  (0.197 to 0.652) | 52 (1 study) | ⊕⊕⊕⊝ **moderate**^1^ |  |
|  | See comment | See comment |  |  |  |  |
|  | **Moderate** | |  |  |  |  |
|  | **750 per 1000** | **391 per 1000** (239 to 595) |  |  |  |  |
| *The basis for the **assumed risk** (e.g. the median control group risk across studies) is provided in footnotes. The **corresponding risk** (and its 95% confidence interval) is based on the assumed risk in the comparison group and the **relative effect** of the intervention (and its 95% CI).  **CI:** Confidence interval; **HR:** Hazard ratio; | | | | | | |
| GRADE Working Group grades of evidence **High quality:** Further research is very unlikely to change our confidence in the estimate of effect.  **Moderate quality:** Further research is likely to have an important impact on our confidence in the estimate of effect and may change the estimate. **Low quality:** Further research is very likely to have an important impact on our confidence in the estimate of effect and is likely to change the estimate. **Very low quality:** We are very uncertain about the estimate. | | | | | | |
| ^1^ Blinding was not implemented | | | | | | |

| **TACE compared to Hepatectomy alone for HCC with a solitary tumor ≥ 5 cm and MVI** | | | | | | |
| --- | --- | --- | --- | --- | --- | --- |
| **Patient or population:** patients with HCC with a solitary tumor ≥ 5 cm and MVI **Settings:** Sun Yat-sen University Cancer Center **Intervention:** TACE **Comparison:** Hepatectomy alone | | | | | | |
| **Outcomes** | **Illustrative comparative risks* (95% CI)** | | **Relative effect (95% CI)** | **No of Participants (studies)** | **Quality of the evidence (GRADE)** | **Comments** |
|  | Assumed risk | Corresponding risk |  |  |  |  |
|  | **Hepatectomy alone** | **TACE** |  |  |  |  |
| **OS** Follow-up: mean 37.5 months | **Study population** | | **HR 0.68**  (0.48 to 0.97) | 234 (1 study) | ⊕⊕⊕⊝ **moderate**^1^ |  |
|  | See comment | See comment |  |  |  |  |
|  | **Moderate** | |  |  |  |  |
|  | **750 per 1000** | **610 per 1000** (486 to 739) |  |  |  |  |
| **DFS** Follow-up: mean 37.5 | **Study population** | | **HR 0.70**  (0.52 to 0.95) | 234 (1 study) | ⊕⊕⊕⊝ **moderate**^1^ |  |
|  | See comment | See comment |  |  |  |  |
|  | **Moderate** | |  |  |  |  |
|  | **750 per 1000** | **621 per 1000** (514 to 732) |  |  |  |  |
| *The basis for the **assumed risk** (e.g. the median control group risk across studies) is provided in footnotes. The **corresponding risk** (and its 95% confidence interval) is based on the assumed risk in the comparison group and the **relative effect** of the intervention (and its 95% CI).  **CI:** Confidence interval; **HR:** Hazard ratio; | | | | | | |
| GRADE Working Group grades of evidence **High quality:** Further research is very unlikely to change our confidence in the estimate of effect.  **Moderate quality:** Further research is likely to have an important impact on our confidence in the estimate of effect and may change the estimate. **Low quality:** Further research is very likely to have an important impact on our confidence in the estimate of effect and is likely to change the estimate. **Very low quality:** We are very uncertain about the estimate. | | | | | | |
| ^1^ Blinding was not implemented | | | | | | |

| **TACE compared to Hepatectomy alone for HCC intermediate or high risk recurrence factors** | | | | | | |
| --- | --- | --- | --- | --- | --- | --- |
| **Patient or population:** patients with HCC intermediate or high risk recurrence factors **Settings:** Zhongshan Hospita **Intervention:** TACE **Comparison:** Hepatectomy alone | | | | | | |
| **Outcomes** | **Illustrative comparative risks* (95% CI)** | | **Relative effect (95% CI)** | **No of Participants (studies)** | **Quality of the evidence (GRADE)** | **Comments** |
|  | Assumed risk | Corresponding risk |  |  |  |  |
|  | **Hepatectomy alone** | **TACE** |  |  |  |  |
| **OS** Follow-up: mean 44.1 months | **Study population** | | **HR 0.59**  (0.36 to 0.97) | 280 (1 study) | ⊕⊕⊕⊝ **moderate**^1^ |  |
|  | See comment | See comment |  |  |  |  |
|  | **Moderate** | |  |  |  |  |
|  | **750 per 1000** | **559 per 1000** (393 to 739) |  |  |  |  |
| **RFS** Follow-up: mean 44.1 months | **Study population** | | **HR 0.68**  (0.49 to 0.93) | 150 (1 study) | ⊕⊕⊕⊝ **moderate**^1^ |  |
|  | See comment | See comment |  |  |  |  |
|  | **Moderate** | |  |  |  |  |
|  | **750 per 1000** | **610 per 1000** (493 to 725) |  |  |  |  |
| *The basis for the **assumed risk** (e.g. the median control group risk across studies) is provided in footnotes. The **corresponding risk** (and its 95% confidence interval) is based on the assumed risk in the comparison group and the **relative effect** of the intervention (and its 95% CI).  **CI:** Confidence interval; **HR:** Hazard ratio; | | | | | | |
| GRADE Working Group grades of evidence **High quality:** Further research is very unlikely to change our confidence in the estimate of effect.  **Moderate quality:** Further research is likely to have an important impact on our confidence in the estimate of effect and may change the estimate. **Low quality:** Further research is very likely to have an important impact on our confidence in the estimate of effect and is likely to change the estimate. **Very low quality:** We are very uncertain about the estimate. | | | | | | |
| ^1^ Blinding was not implemented | | | | | | |

| **TACE compared to Hepatectomy alone for Stage IIIA HCC** | | | | | | |
| --- | --- | --- | --- | --- | --- | --- |
| **Patient or population:** patients with Stage IIIA HCC **Settings:** Cancer Center of Sun Yat-Sen University **Intervention:** TACE **Comparison:** Hepatectomy alone | | | | | | |
| **Outcomes** | **Illustrative comparative risks* (95% CI)** | | **Relative effect (95% CI)** | **No of Participants (studies)** | **Quality of the evidence (GRADE)** | **Comments** |
|  | Assumed risk | Corresponding risk |  |  |  |  |
|  | **Hepatectomy alone** | **TACE** |  |  |  |  |
| **OS** Follow-up: mean 20 months | **Study population** | | **HR 0.72**  (0.47 to 1.10) | 115 (1 study) | ⊕⊕⊕⊝ **moderate**^1^ |  |
|  | See comment | See comment |  |  |  |  |
|  | **Moderate** | |  |  |  |  |
|  | **750 per 1000** | **631 per 1000** (479 to 782) |  |  |  |  |
| **DFS** Follow-up: mean 20 months | **Study population** | | **HR 0.55**  (0.37 to 0.82) | 115 (1 study) | ⊕⊕⊕⊝ **moderate**^1^ |  |
|  | See comment | See comment |  |  |  |  |
|  | **Moderate** | |  |  |  |  |
|  | **750 per 1000** | **533 per 1000** (401 to 679) |  |  |  |  |
| *The basis for the **assumed risk** (e.g. the median control group risk across studies) is provided in footnotes. The **corresponding risk** (and its 95% confidence interval) is based on the assumed risk in the comparison group and the **relative effect** of the intervention (and its 95% CI).  **CI:** Confidence interval; **HR:** Hazard ratio; | | | | | | |
| GRADE Working Group grades of evidence **High quality:** Further research is very unlikely to change our confidence in the estimate of effect.  **Moderate quality:** Further research is likely to have an important impact on our confidence in the estimate of effect and may change the estimate. **Low quality:** Further research is very likely to have an important impact on our confidence in the estimate of effect and is likely to change the estimate. **Very low quality:** We are very uncertain about the estimate. | | | | | | |
| ^1^ Blinding was not implemented | | | | | | |

| **TACE compared to Hepatectomy alone for HCC with PVTT** | | | | | | |
| --- | --- | --- | --- | --- | --- | --- |
| **Patient or population:** patients with HCC with PVTT **Settings:** The First Affiliated Hospital of Sun Yat-Sen University **Intervention:** TACE **Comparison:** Hepatectomy alone | | | | | | |
| **Outcomes** | **Illustrative comparative risks* (95% CI)** | | **Relative effect (95% CI)** | **No of Participants (studies)** | **Quality of the evidence (GRADE)** | **Comments** |
|  | Assumed risk | Corresponding risk |  |  |  |  |
|  | **Hepatectomy alone** | **TACE** |  |  |  |  |
| **OS** Follow-up: median 33.6 months | **Study population** | | **HR 0.61**  (0.30 to 0.98) | 104 (1 study) | ⊕⊕⊕⊝ **moderate**^1^ |  |
|  | See comment | See comment |  |  |  |  |
|  | **Moderate** | |  |  |  |  |
|  | **750 per 1000** | **571 per 1000** (340 to 743) |  |  |  |  |
| *The basis for the **assumed risk** (e.g. the median control group risk across studies) is provided in footnotes. The **corresponding risk** (and its 95% confidence interval) is based on the assumed risk in the comparison group and the **relative effect** of the intervention (and its 95% CI).  **CI:** Confidence interval; **HR:** Hazard ratio; | | | | | | |
| GRADE Working Group grades of evidence **High quality:** Further research is very unlikely to change our confidence in the estimate of effect.  **Moderate quality:** Further research is likely to have an important impact on our confidence in the estimate of effect and may change the estimate. **Low quality:** Further research is very likely to have an important impact on our confidence in the estimate of effect and is likely to change the estimate. **Very low quality:** We are very uncertain about the estimate. | | | | | | |
| ^1^ Blinding was not implemented | | | | | | |
