# Supplementary material for: Comparative effectiveness of adjuvant treatment for hepatocellular carcinoma with high risk of recurrence: A systematic review and network meta-analysis
Source: PLoS One. 2025 Dec 4;20(12):e0335457. doi: 10.1371/journal.pone.0335457 (PMC12677550; doi:10.1371/journal.pone.0335457)
Supplement: S1 File — (ZIP) [file pone.0335457.s001.zip › Supplementary Material/S6 File.docx]

| Study | Year | PMID | 1 | 2 | 3 | 4 | 5 | 6 | 7 | Overall bias |
| --- | --- | --- | --- | --- | --- | --- | --- | --- | --- | --- |
| Xiang | 2024 | 37812183 | Moderate | Low | Low | Moderate | Moderate | Low | Low | Moderate |
| Luo | 2023 | 36905230 | Moderate | Low | Low | Moderate | Modetate | Low | Low | Moderate |
| Long | 2023 | 36634853 | Moderate | Low | Low | Moderate | Modetate | Low | Low | Moderate |
| Li | 2023 | 37452107 | Moderate | Moderate | Low | Low | Low | Low | Low | Moderate |
| Li | 2023 | 37359534 | Moderate | Moderate | Low | Low | Modetate | Low | Low | Moderate |
| Bai | 2023 | 37029989 | Moderate | Moderate | Low | Low | Low | Low | Low | Moderate |
| Gou | 2022 | 35643251 | Moderate | Moderate | Low | Low | Low | Low | Low | Moderate |
| Lin | 2022 | 35300207 | Moderate | Moderate | Low | Low | Low | Low | Low | Moderate |
| Qiu | 2022 | 35795039 | Moderate | Moderate | Low | Low | Low | Low | Low | Moderate |
| Wang | 2021 | 33455865 | Moderate | Moderate | Low | Low | Low | Low | Low | Moderate |
| Li | 2021 | 34631511 | Moderate | Moderate | Low | Low | Low | Low | Low | Moderate |
| Wang | 2021 | 32440804 | Moderate | Moderate | Low | Low | Low | Low | Low | Moderate |
| Huang | 2020 | 33061610 | Moderate | Moderate | Low | Low | Low | Low | Low | Moderate |
| Wang | 2020 | 32611327 | Moderate | Moderate | Low | Low | Low | Low | Low | Moderate |
| Wang | 2020 | 32547217 | Moderate | Moderate | Low | Low | Low | Low | Low | Moderate |
| Zhang | 2019 | 30767178 | Moderate | Moderate | Low | Low | Low | Low | Low | Moderate |
| Zhang | 2019 | 31153833 | Moderate | Moderate | Low | Low | Low | Low | Low | Moderate |
| Wang | 2019 | 30863091 | Moderate | Moderate | Moderate | Low | Low | Low | Low | Moderate |
| Qi | 2019 | 30103903 | Moderate | Moderate | Low | Low | Low | Low | Low | Moderate |
| Wang | 2019 | 30249510 | Moderate | Moderate | Low | Low | Low | Moderate | Low | Moderate |
| Li | 2017 | 28032575 | Moderate | Moderate | Low | Low | Low | Moderate | Low | Moderate |
| Hsiao | 2017 | 28728985 | Moderate | Moderate | Low | Moderate | Low | Moderate | Low | Moderate |
| Xia | 2016 | 27340354 | Moderate | Moderate | Low | Moderate | Moderate | Moderate | Low | Moderate |
| Li | 2015 | 24972992 | Moderate | Moderate | Low | Moderate | Moderate | Moderate | Low | Moderate |

1:Bias due to confounding;2:Bias in selection of participants into the study;3:Bias in classification of interventions;4:Bias due to deviations from intended interventions;5:Bias due to missing data;6:Bias in measurement of outcomes;7:Bias in selection of the reported result
