# Supplementary figures and images for: Comparative effectiveness of adjuvant treatment for hepatocellular carcinoma with high risk of recurrence: A systematic review and network meta-analysis
Source: PLoS One. 2025 Dec 4;20(12):e0335457. doi: 10.1371/journal.pone.0335457 (PMC12677550; doi:10.1371/journal.pone.0335457)

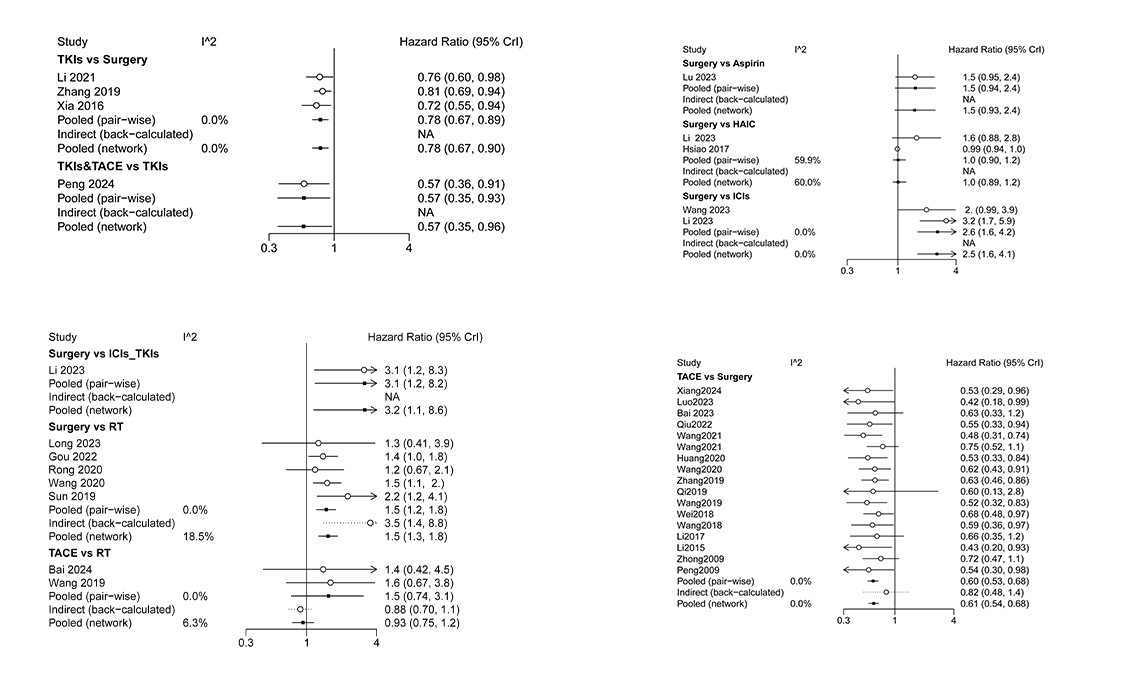

Supplement: S1 File — (ZIP) [file pone.0335457.s001.zip › Supplementary Material/S11 File.png]

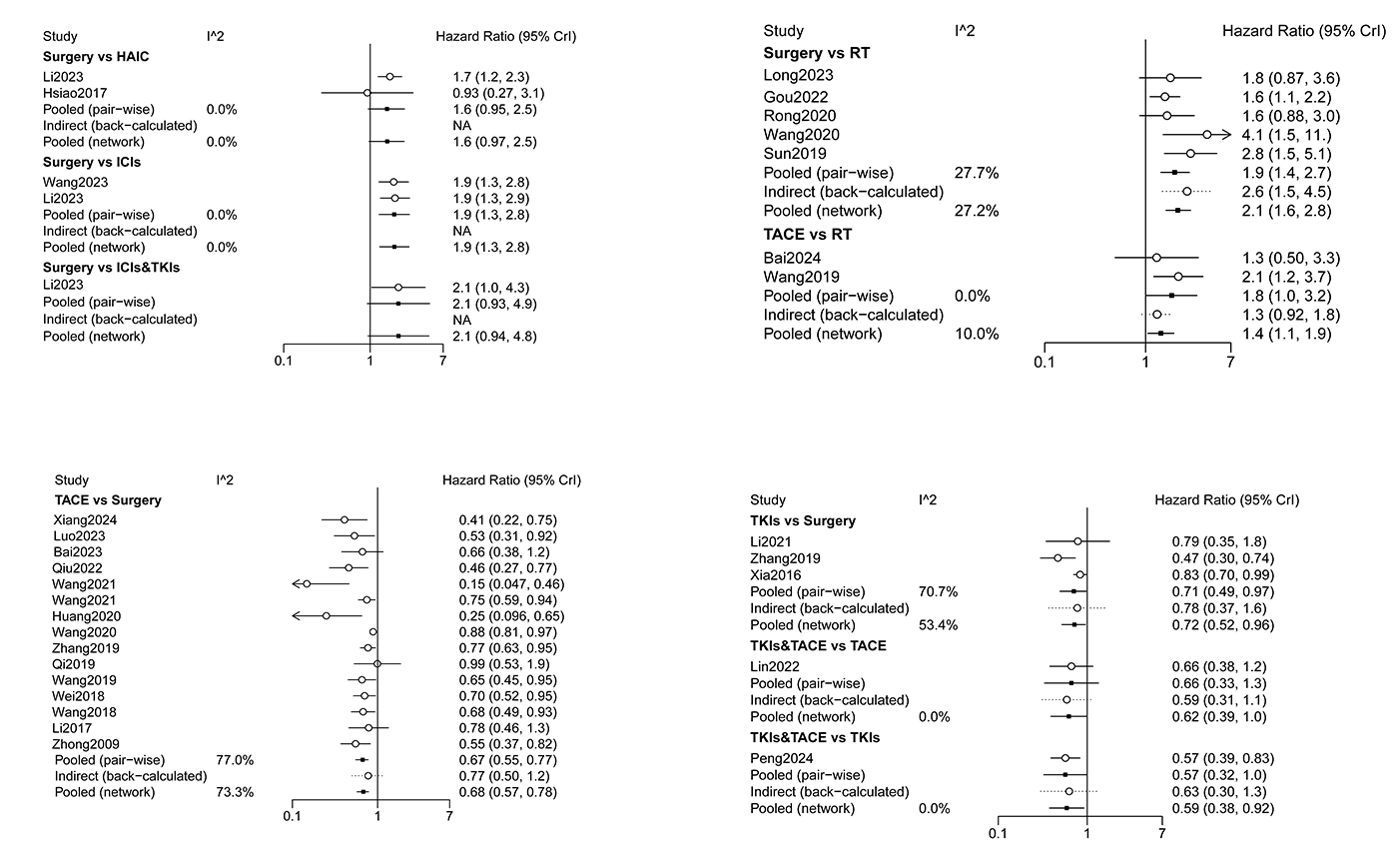

Supplement: S1 File — (ZIP) [file pone.0335457.s001.zip › Supplementary Material/S12 File.png]

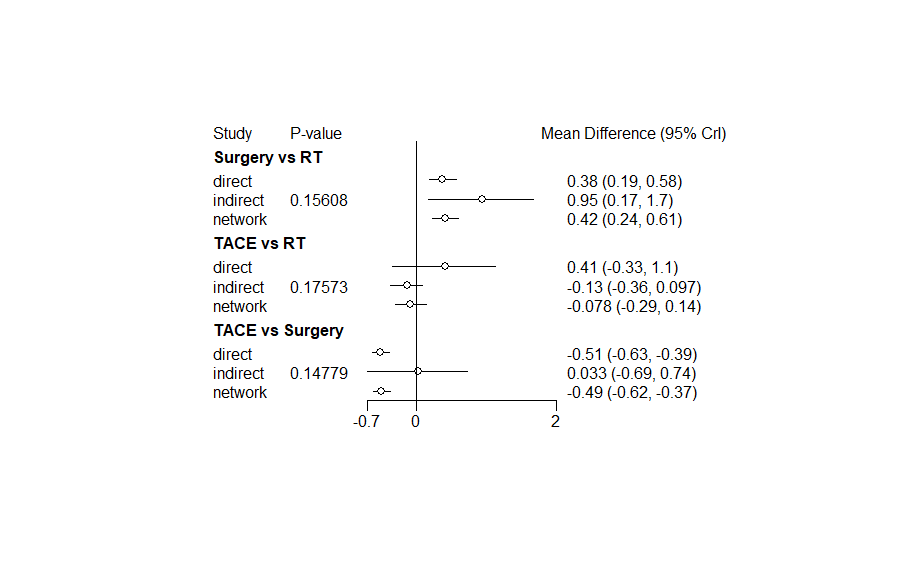

Supplement: S1 File — (ZIP) [file pone.0335457.s001.zip › Supplementary Material/S13 File.png]

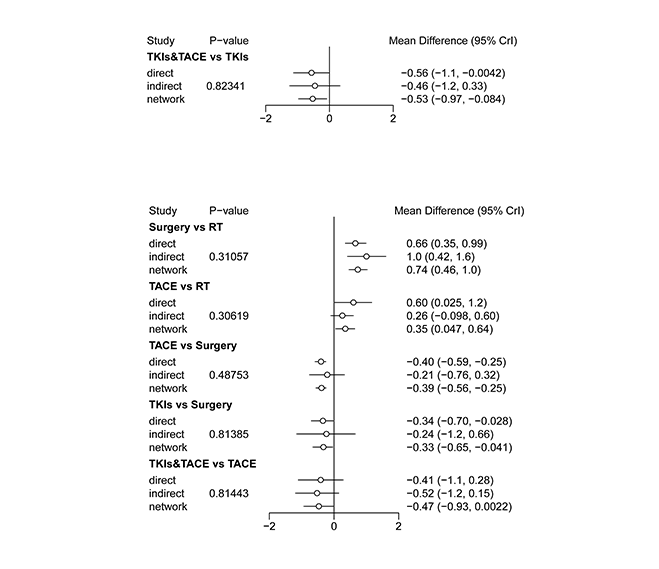

Supplement: S1 File — (ZIP) [file pone.0335457.s001.zip › Supplementary Material/S14 File.png]

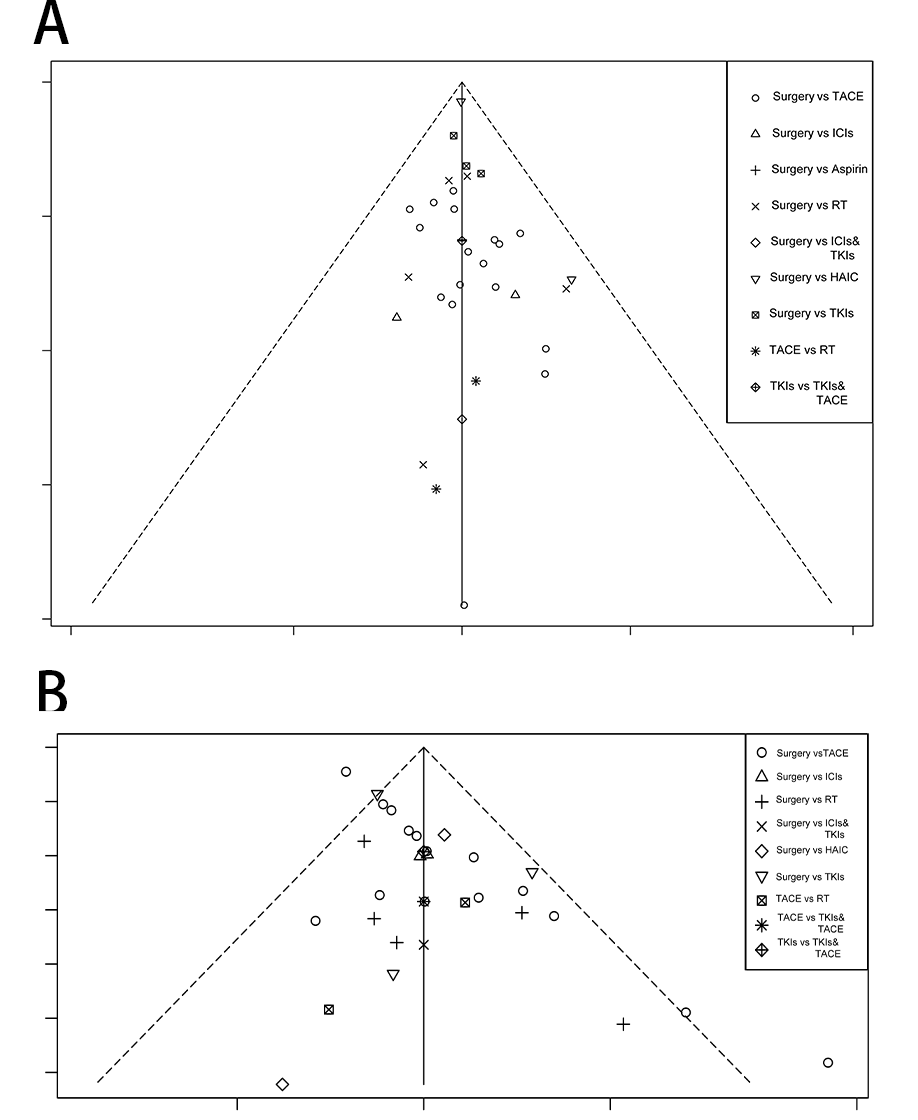

Supplement: S1 File — (ZIP) [file pone.0335457.s001.zip › Supplementary Material/S15 File.png]

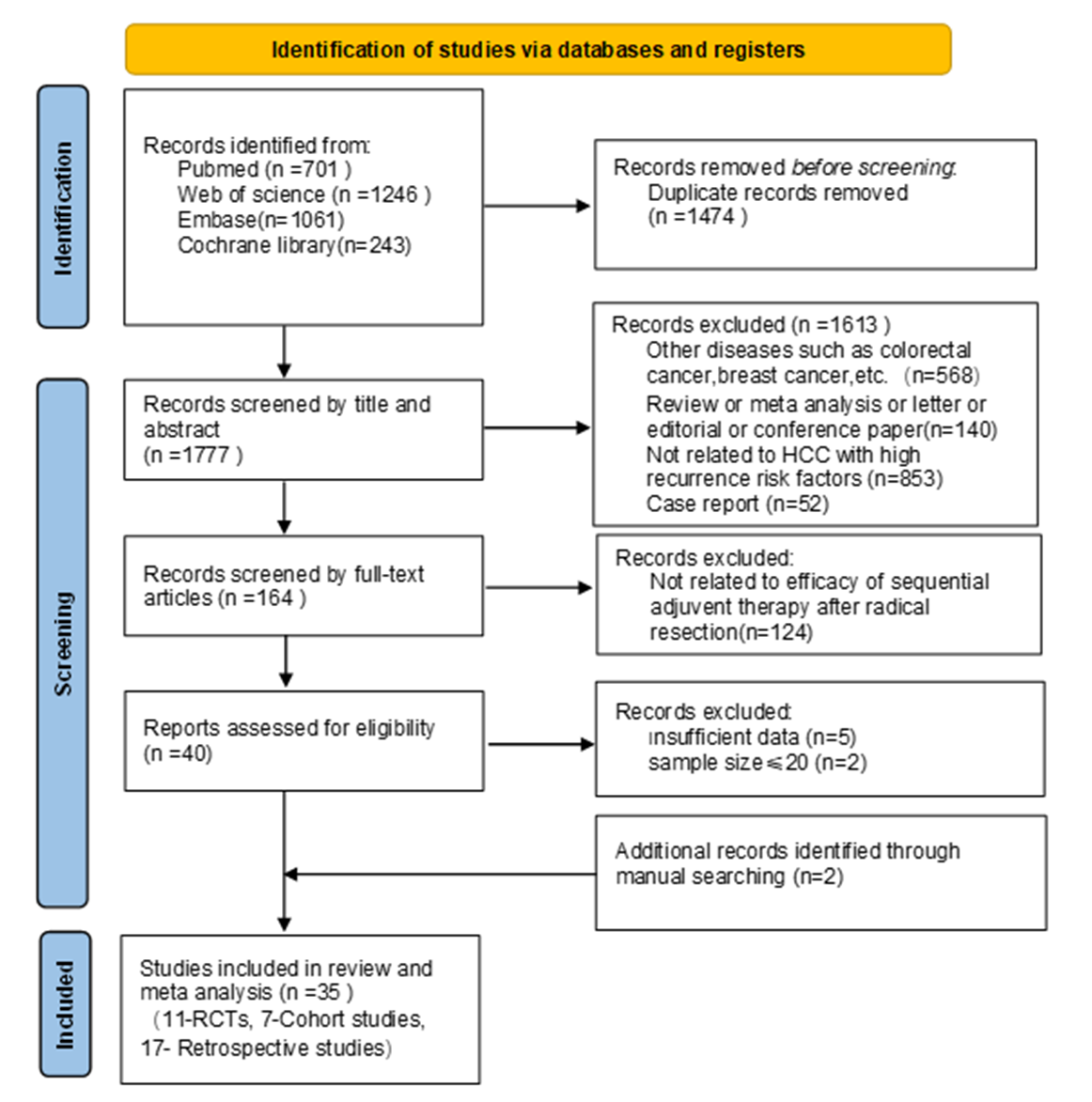

Supplement: S1 File — (ZIP) [file pone.0335457.s001.zip › Supplementary Material/S2 File.tif]

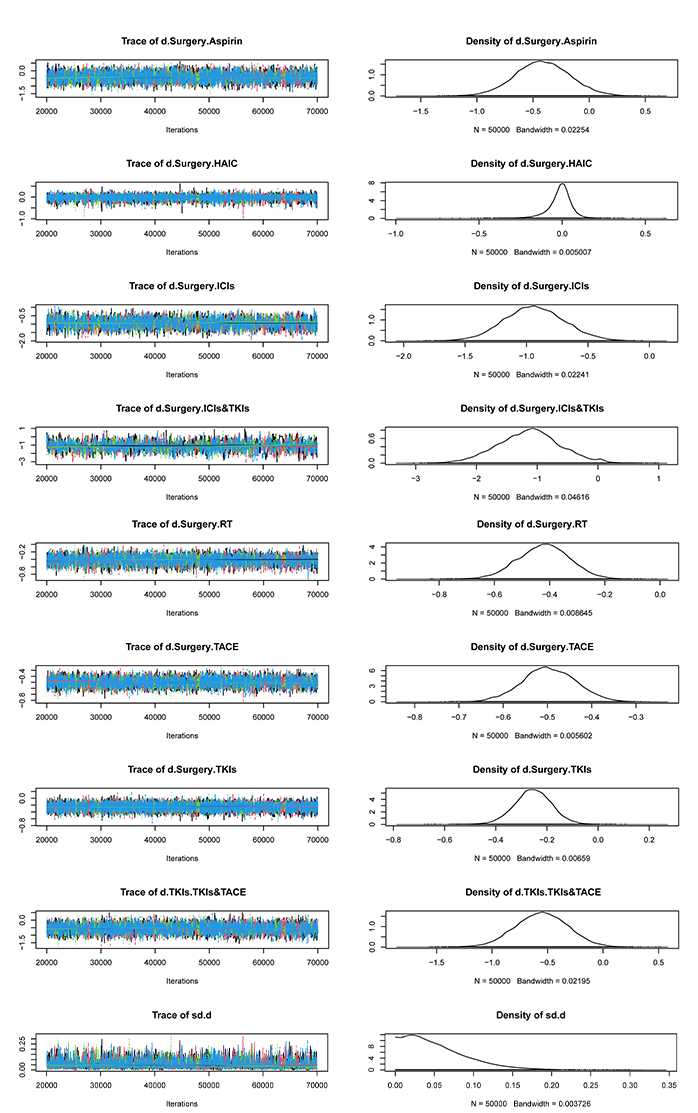

Supplement: S1 File — (ZIP) [file pone.0335457.s001.zip › Supplementary Material/S7 File.png]

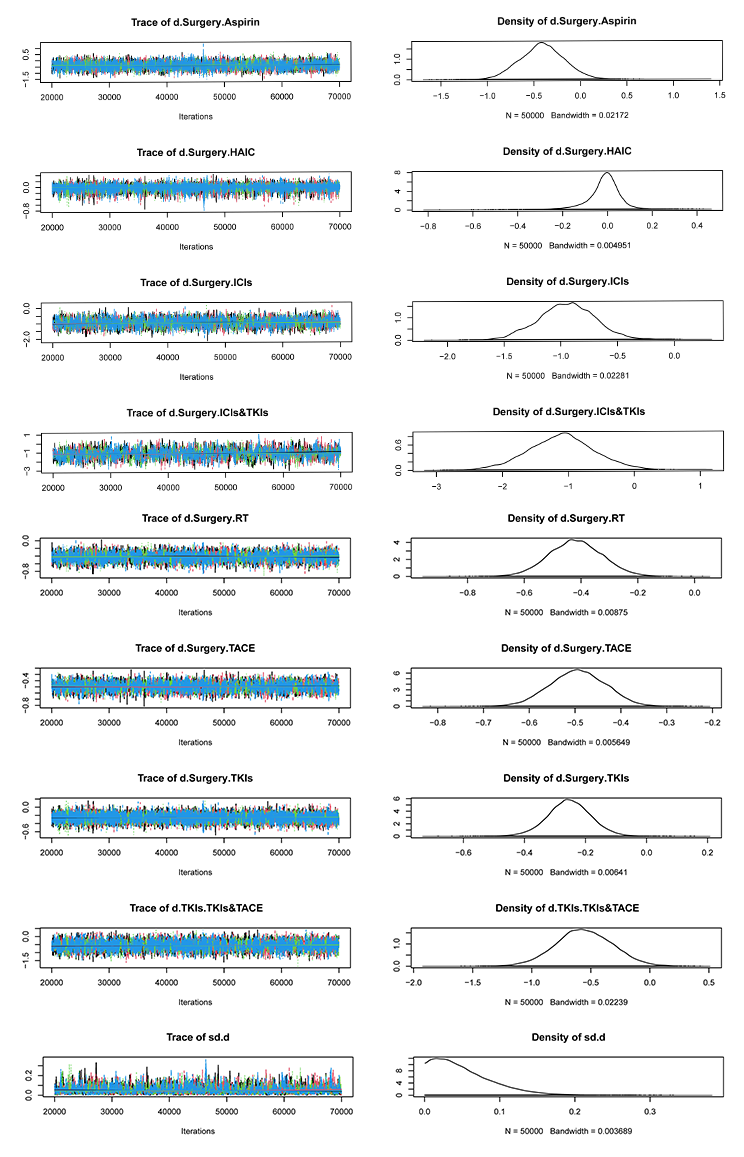

Supplement: S1 File — (ZIP) [file pone.0335457.s001.zip › Supplementary Material/S8 File.png]
